# Maintenance and dissemination of avian-origin influenza A virus within the northern Atlantic Flyway of North America

**DOI:** 10.1371/journal.ppat.1010605

**Published:** 2022-06-06

**Authors:** Diann J. Prosser, Jiani Chen, Christina A. Ahlstrom, Andrew B. Reeves, Rebecca L. Poulson, Jeffery D. Sullivan, Daniel McAuley, Carl R. Callahan, Peter C. McGowan, Justin Bahl, David E. Stallknecht, Andrew M. Ramey

**Affiliations:** 1 U.S. Geological Survey, Eastern Ecological Science Center, Laurel, Maryland, United States of America; 2 Center for Ecology of Infectious Diseases, Department of Infectious Diseases, Department of Epidemiology and Biostatistics, Institute of Bioinformatics, University of Georgia, Athens, Georgia, United States of America; 3 U.S. Geological Survey, Alaska Science Center, Anchorage, Alaska, United States of America; 4 U.S. Geological Survey, National Wildlife Health Center, Madison, Wisconsin, United States of America; 5 Southeastern Cooperative Wildlife Disease Study, Department of Population Health, College of Veterinary Medicine, University of Georgia, Athens, Georgia, United States of America; 6 U.S. Fish and Wildlife Service, Chesapeake Bay Field Office, Annapolis, Maryland, United States of America; Erasmus Medical Center, NETHERLANDS

## Abstract

Wild waterbirds, the natural reservoirs for avian influenza viruses, undergo migratory movements each year, connecting breeding and wintering grounds within broad corridors known as flyways. In a continental or global view, the study of virus movements within and across flyways is important to understanding virus diversity, evolution, and movement. From 2015 to 2017, we sampled waterfowl from breeding (Maine) and wintering (Maryland) areas within the Atlantic Flyway (AF) along the east coast of North America to investigate the spatio-temporal trends in persistence and spread of influenza A viruses (IAV). We isolated 109 IAVs from 1,821 cloacal / oropharyngeal samples targeting mallards *(Anas platyrhynchos)* and American black ducks *(Anas rubripes)*, two species having ecological and conservation importance in the flyway that are also host reservoirs of IAV. Isolates with >99% nucleotide similarity at all gene segments were found between eight pairs of birds in the northern site across years, indicating some degree of stability among genome constellations and the possibility of environmental persistence. No movement of whole genome constellations were identified between the two parts of the flyway, however, virus gene flow between the northern and southern study locations was evident. Examination of banding records indicate direct migratory waterfowl movements between the two locations within an annual season, providing a mechanism for the inferred viral gene flow. Bayesian phylogenetic analyses provided evidence for virus dissemination from other North American wild birds to AF dabbling ducks (Anatinae), shorebirds (Charidriformes), and poultry (Galliformes). Evidence was found for virus dissemination from shorebirds to gulls (Laridae), and dabbling ducks to shorebirds and poultry. The findings from this study contribute to the understanding of IAV ecology in waterfowl within the AF.

## Author summary

The recent first detection of highly pathogenic avian influenza (HPAI) virus in wild birds inhabiting the Atlantic Flyway of North America (Newfoundland, Canada in November 2021 and South Carolina, USA, in January 2022) raises concern for virus movement within this region. Here, we study IAV in wild waterfowl with focus on bird movements in combination with IAV prevalence, seroprevalence, and genetic analyses for hypothesized virus flow and persistence. Migratory connectivity for two important dabbling duck species within the AF was indicated by banding records from the North American Bird Banding Program. A broad diversity of viral subtypes including H1, H3, H4, H6, H8–11, N1–4, N6, N8–N9 was detected in these species. Prevalence of active infections was higher on the breeding than wintering grounds, and common to other studies, the reverse was seen for antibody prevalence. Genetic analysis indicated the possibility of virus persistence from one year to the next in the northern latitudes on the breeding grounds, and although no full genome movement was detected between the breeding and wintering sites, viral gene flow was evident. The contribution of these findings in relation to bird movements will help guide surveillance as HPAI is detected within the flyway.

## Introduction

Wild waterfowl (Anatidae) are a primary natural reservoir for low pathogenic (LP) influenza A viruses (IAV), serving an important role in viral persistence, amplification, and spread [1]. Most waterfowl species are highly mobile across the annual cycle, migrating hundreds to thousands of kilometers between breeding and wintering grounds [2]. These annual cycle movements can have important consequences for avian influenza virus spread and risk to susceptible populations, be it other wildlife species [3,4] or economically important agriculture systems such as poultry production [5–7].

Patterns of virus transmission depend on complex relationships between seasonal migrations, subsequent interactions among populations, and characteristics of host immunity and susceptibility. For example, susceptibility differs across ages, with immunologically naïve juveniles having higher susceptibility than adults [8]. Viral dynamics vary across species, with even closely related species expressing differences in susceptibility, asymptomatic periods, virus shedding rates and duration, clinical signs, and mortality [9–11]. Additionally, virus subtype diversity can change seasonally as birds migrate from breeding to wintering grounds, due to changes in population immunity as the season progresses [12–15], and as populations mix at stopover sites along migration [16] or on the wintering grounds [4,17,18]. The ability of viruses to persist for weeks to months in moist or aquatic environments adds to the complexity of virus exposure and movement by the migratory wild hosts [18–21]. Collectively, patterns of higher prevalence have been detected on northern breeding grounds followed by a reduction in prevalence and increase in virus diversity during the migration and wintering periods [8,22,23] within the migratory corridors [24–26]. Thus, understanding the dynamics of avian influenza across space and time for different species requires understanding the movements of migratory birds, persistence of the virus in the environment, frequency of transmission across species, and differences in prevalence or susceptibility.

A flyway perspective based on broad migratory corridors for avian species, particularly waterfowl, has long been implemented for ecological study and management [27,28]. In the context of IAV movement by wild waterfowl, surveillance plans [29,30] and research studies [24,25,31] have considered a flyway perspective, with additional focus put on pathways believed to have higher inter-continental wild bird connectivity. For example, research has been dedicated to understanding transmission dynamics in the Pacific Flyway, the migratory corridor that links western North America with eastern regions of Asia via breeding grounds in Beringia [32,33], due in part to the concern that avian influenza viruses may be introduced or passed between North America and Asia [34,35]. A key example of such an introduction occurred in 2014 when Asian-origin highly pathogenic (HP) IAV H5N8 entered North America during the fall migration [36], reassorted, and spread throughout the Pacific and Central Flyways of the U.S. where it was introduced to domestic poultry facilities and affected over 50 million poultry prior to suspected eradication in June 2015 [36]. Concern for HPAI entering North America from Europe via the Atlantic Flyway has been raised with less attention [37], however, recent detection of HPAI H5N1 in Newfoundland Canada [38,39] and subsequent spread to all four North American flyways [40–42] has confirmed the ecological and economical relevance of this viral dissemination pathway.

Introduction of HPAI into the Atlantic Flyway is concerning given the importance of this region to migratory waterfowl and domestic poultry production. Following breeding, waterfowl from the northern part of the flyway migrate to wintering grounds such as the Chesapeake Bay [43,44], which is recognized as an important wintering and stopover habitat [45,46] for several waterfowl species such as the mallard (*Anas platyrhynchos*) and American black duck (*Anas rubripes*; hereafter black duck) [47,48]. The extensive poultry production that occurs on the Delmarva Peninsula [49], which borders a large portion of the Chesapeake Bay, provides a significant potential interface for viral transmission between wild and domestic bird populations. This combination of domestic poultry, concentrated waterfowl, and potential for introductions of novel influenza strains into these populations presents a need for improved understanding of IAV ecology in the Atlantic Flyway.

The goal of this study was to increase our understanding of the spatiotemporal trends in occurrence of avian influenza viruses within dabbling ducks of the Atlantic Flyway and how these trends affect the persistence and spread of virus at multiple scales. To address this broad goal, our objectives were to (1) explore the migratory connectivity of waterfowl between a northern breeding site (Maine) and an important stopover and wintering habitat in the Atlantic Flyway (the Chesapeake Bay, Maryland), (2) identify trends in seroprevalence and viral strains detected in birds from these locations, and (3) use genomic information for influenza virus isolates to investigate (a) potential viral persistence within the region, (b) dispersal between two locations, and (c) virus dissemination across a suite of hosts. Addressing these questions will allow for an improved understanding of the ecology of avian influenza in this important migratory flyway and across latitudinal gradients.

## Results

### Waterfowl sampling and bird banding connections

We collected and analyzed 1821 paired swab and 1031 serum samples in this study. While mallards and black ducks represented the majority of our analyzed swab (1646) and serum (969) samples, other dabbling duck species (northern pintail, *Anas acuta;* green-winged teal, *Anas carolinensis;* blue-winged teal, *Spatula discors;* gadwall, *Mareca strepera;* northern shoveler, *Spatula clypeata*) were included opportunistically (S1 Table). We collected 155 swab samples from hunter harvested birds, 46 of which were from either mallards or black ducks (S1 Table). We collected a total of 762 and 904 swabs from live-captured birds in Maine and Maryland, respectively, of which mallards and black ducks constituted 759 (99.6%) and 841 (93.0%) of the swab samples analyzed. Similarly, while serum samples were collected from 253 and 791 of the birds captured in Maine and Maryland, respectively, only 242 and 789 were included in final analyses following removal of samples with insufficient volumes of serum. Of the mallard and black duck serum samples included in final analyses, 242 were from Maine and 727 were from Maryland.

**Fig 1:**
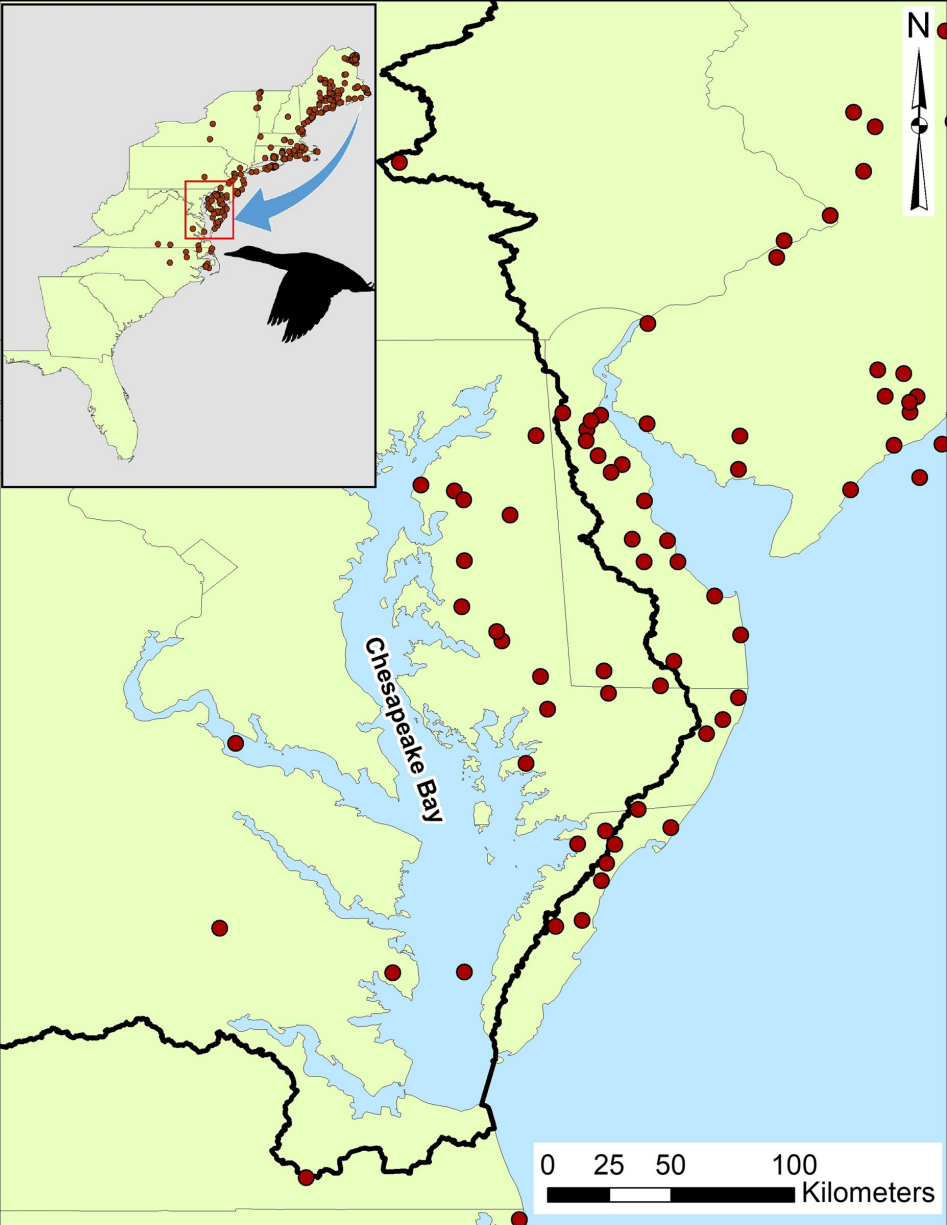
Waterfowl migratory connectivity. Within-season migratory connections between sampling areas within the North Atlantic migratory flyway (Maine, Maryland, respectively) shown as re-encounter locations for two primary waterfowl species: mallard and American black duck. Data obtained for a 10-year period (2011 through 2021) from the North American Bird Banding Program. Red dots indicate birds banded in Maine and re-encountered in the same migratory season (for example, potentially bred in Maine and wintered in Maryland). The black line indicates the extent of the Chesapeake Bay watershed. Inset shows reencounter locations across the entire region (Maine through North Carolina, USA) and main panel is zoomed to the wintering locations near the Chesapeake Bay. The base layer was downloaded from the U.S. Census Bureau (https://www.census.gov/geographies/mapping-files/time-series/geo/carto-boundary-file.html) and used as part of the public domain.

Banding data from the U.S. Geological Survey Bird Banding Lab (hereafter BBL) database included 21 mallards and black ducks that were banded in Maine and encountered in the Maryland and Virginia portions of the Chesapeake Bay watershed within the same migratory cycle. These observations were concentrated along the Eastern Shore of Maryland and occurred across numerous years (Fig 1). Although the BBL database shows migratory connectivity between these sites, unsurprisingly, none of the birds sampled for IAV and marked in this study were observed in both Maine and Maryland.

### Virus prevalence and antibody seroprevalence

Detections of IAV via rRT-PCR varied across sampling locations and years (S2 Table). On average, inferred prevalence (matrix protein, M+) varied across species, however when controlling for state and year, there were no species-level pairwise differences (p > 0.05). These findings may have been driven by the limited sample size for species other than mallard and black duck. Across the flyway, prevalence was higher in Maine where birds were sampled on the breeding grounds, than in Maryland, where birds were sampled during fall migration and early winter (p < 0.001; SE = 0.13; Table 1). An example of this trend is evidenced by data for year 2016 when waterfowl sampled in Maine showed highest prevalence across sites and years (48% as inferred using rRT-PCR, lowest prevalence across sites and years was 14%; p < 0.001; SE = 0. 13). Virus isolation rates (VI/M+ ratios) were also higher in Maine than Maryland (p < 0.001; SE = 0.26).

**Table 1:** Prevalence and seroprevalence of influenza A virus for wild dabbling ducks sampled in Maine and Maryland from 2015–2017. Prevalence identified using rRT-PCR (M+, Ct values ≤ 45) and virus isolation (VI). Seroprevalence (avian influenza antibodies) identified using bELISA with serum-to-negative control (S/N) ratios <0.5 and 0.7. Dashes indicate no data was collected.

	Prevalence (Ct < = 45)						Seroprevalence		
State	n	M+	VI	M+ Prevalence	VI Prevalence	VI/M+	n	% Positive (S/N < 0.5)	% Positive (S/N < 0.7)
Maine									
2015	220	44	16	20%	7%	36%	126	42%	69%
2016	266	128	30	48%	11%	23%	116	17%	32%
2017	276	75	34	27%	12%	46%	-	-	-
Total	762	247	80	32%	11%	33%	242	30%	51%
Maryland									
2015	578	82	14	14%	2%	17%	481	49%	73%
2016	481	74	15	15%	3%	21%	308	56%	75%
Total	1059	156	29	15%	3%	19%	789	52%	74%

While antibody seroprevalence also varied by sampling location and year (range 17 to 56% and 32 to 75% using S/N thresholds of < 0.5, and 0.7, respectively; Table 1), these values had opposite trends to those seen for active viral infection, with higher seroprevalence on the Maryland wintering grounds than at the Maine breeding sites (Table 1; p < 0.001; SE = 0.09 and 0.15, S/N 0.5 and 0.7, respectively). Seroprevalence generally did not vary between species (p > 0.05) with the only exceptions being that seroprevalence at the S/N < 0.7 threshold was lower in northern pintail than both mallard (p = 0.005, SE = 0.29) and black duck (p = 0.020, SE = 0.31). However, as with the lack of species-specific differences in prevalence, the limited differences in seroprevalence may be driven by sample size. Raw data supporting all virus isolation and seroprevalence results are available at USGS ScienceBase Repository: https://doi.org/10.5066/P9UF27FI.

### RNA extraction and genetic sequencing

**Fig 2:**
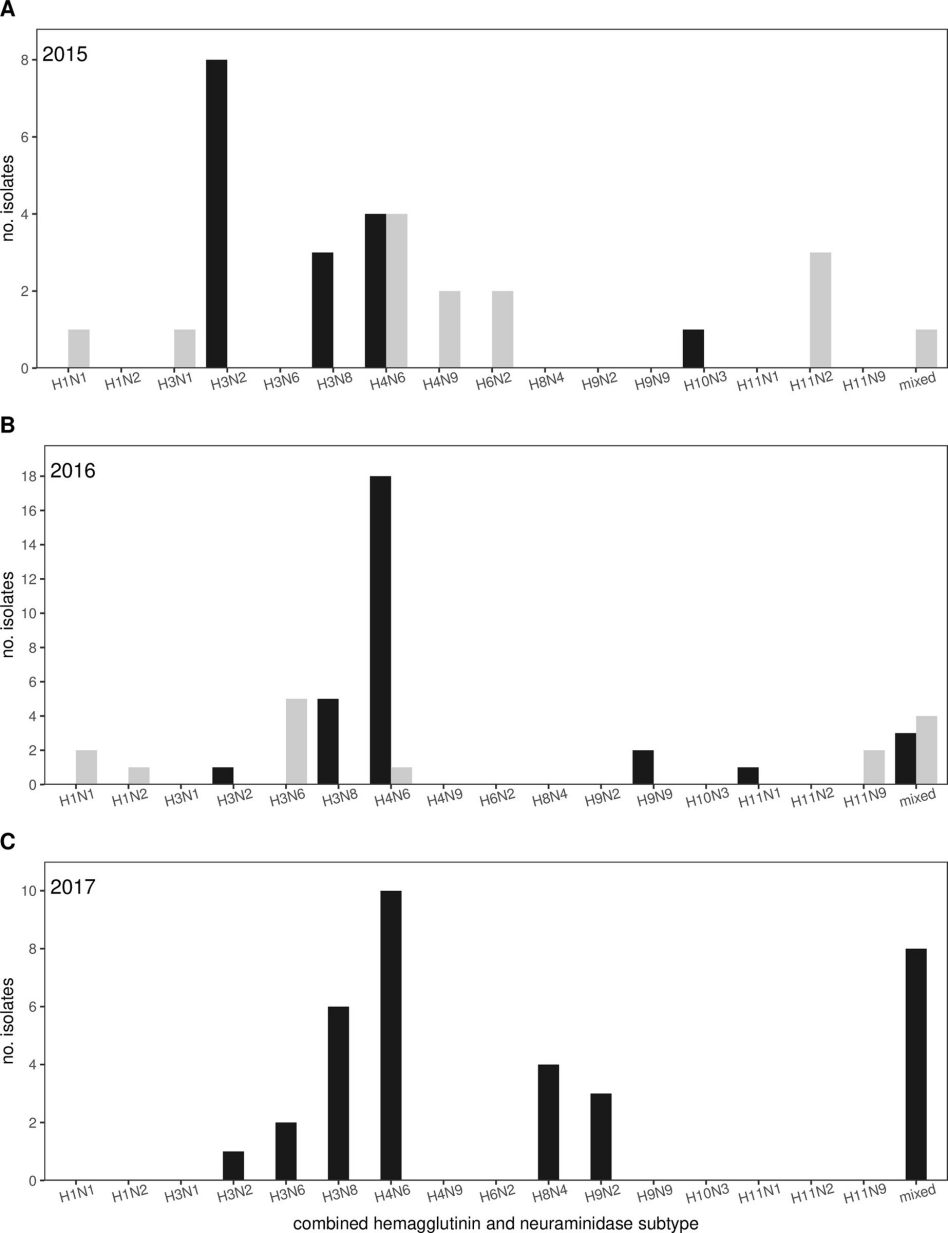
Influenza A virus subtypes detected in this study. Bar charts depicting combined subtypes of influenza A virus (IAV) isolates recovered from samples collected at study sites in Maine (black bars) or Maryland (gray bars) in 2015 (Panel A), 2016 (Panel B), and 2017 (Panel C).

We successfully sequenced genomes of 109 influenza A virus isolates derived from paired cloacal and oropharyngeal swabs collected from dabbling ducks sampled in Maine and Maryland during 2015 (n = 30), 2016 (n = 45), and 2017 (Maine only; n = 34). Viruses were of 16 combined HA and NA subtypes (Fig 2), the most common of which, H4N6, was detected in samples collected from both states and in every year. Viruses of the H3N2 and H3N8 subtypes were also isolated from wild bird samples collected in Maine in all three sample years. We identified a total of 16 mixed infections among the 109 isolates sequenced (14.7%), including at least one mixed infection from each sample year.

### Assessment of viral persistence and dispersal

In comparing genomic sequences obtained from wild bird samples collected in Maine and Maryland during 2015–2017, we identified 87 pairwise comparisons of viruses from the same state and the same year (of 1043 possible comparisons) that shared > 99% nucleotide identity at all eight gene segments (S1 File). We also identified two H3N8 isolates from Maine 2016 samples that shared >99% identity at all eight gene segments with four H3N8 isolates from Maine 2017 samples (eight of 1868 pairwise comparisons). These included H3N8 viruses from two mallards sampled in Maine in 2016 (A/mallard/Maine/UGAI16-5406/2016 (H3N8) and A/mallard/Maine/UGAI16-5418/2016 (H3N8)) in comparison to four birds sampled in Maine in 2017: three mallards and one black duck (A/mallard/Maine/ME-Y17-157/2017 (H3N8), A/mallard/Maine/ME-Y17-165/2017 (H3N8), A/mallard/Maine/ME-Y17-166/2017 (H3N8), A/American black duck/Maine/ME-Y17-170/2017 (H3N8)); S1 File). No isolates from Maine and Maryland shared > 99% nucleotide identity at all eight gene segments, providing no evidence of direct dispersal of whole-genome viral constellations between these two ends of the northern Atlantic Flyway. We did, however, identify many pairwise comparisons of virus sequences from different years and different states that shared > 99% nucleotide identity at one to seven gene segments, providing evidence of viral gene flow between locations (494 of 2240 pairwise comparisons; S1 File).

### Assessment of viral dissemination among diverse avian hosts

**Fig 3:**
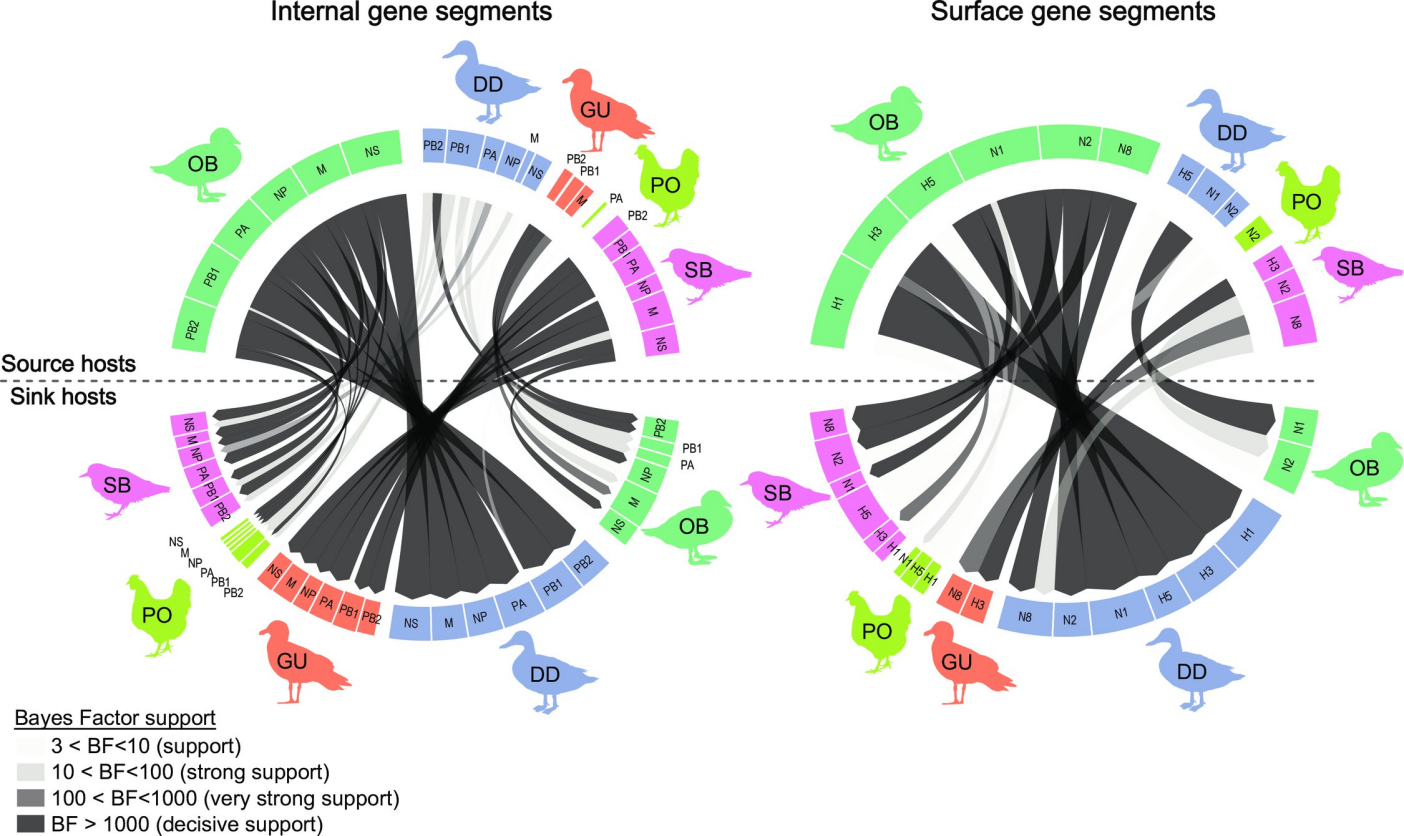
Virus dissemination of gene segments for host groups. Chord diagrams depicting Bayes factors (BF) for viral dissemination among northern Atlantic Flyway dabbling duck (DD), northern Atlantic Flyway shorebird (SB), northern Atlantic Flyway gull (GU), northern Atlantic Flyway poultry (PO), and other North American wild bird (OB) host groups. Inferred host and sink host groups are depicted above and below the horizontal dashed line, respectively. Chord width is proportional to the median transition rate. Statistically supported Bayes Factor (BF > 3.0) are depicted with shaded arrows with the strength of statistical support increasing with intensity of shading. Silhouettes were downloaded from PhyloPic (http://phylopic.org/) and used with permissions granted under Creative Commons Attribution 3.0 Unported license (https://creativecommons.org/licenses/by/3.0/deed.en) or as part of the public domain with images attributed to Rebecca Groom and Sharon Wegner-Larsen.

**Fig 4:**
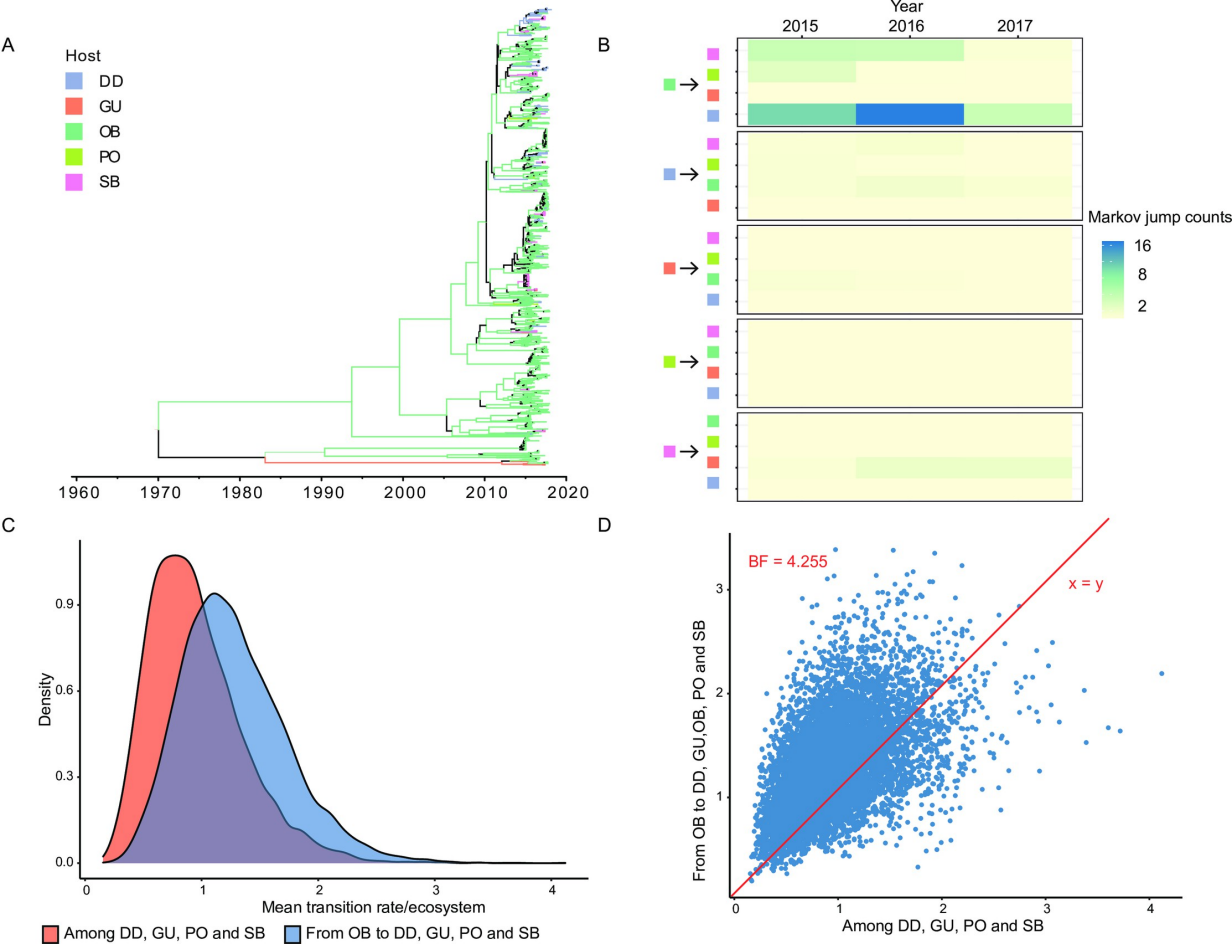
PB2 gene segment phylogenetic tree and Markov jumps. Bayesian phylogenetic tree (Panel A), heat map inferred number of Markov jumps among hosts groups (Panel B), plot of estimates for transitions from the other North American wild bird host group to northern Atlantic Flyway host groups as compared to strictly among northern Atlantic Flyway host groups (Panel C), and plot of ratios of posterior odds versus prior odds to infer differences in migration rate estimates between the other North American wild bird host group and northern Atlantic Flyway host groups as compared to strictly among northern Atlantic Flyway host groups (Panel D) for the PB2 gene segment. Comparable information for other 7 gene segments is provided in S2 File. Entire phylogenetic trees (e.g., Panel A) are provided in S4 File as PDFs and S5 File as TreeTaxa plots. Abbreviations for hosts groups are as follows: DD = northern Atlantic Flyway dabbling duck, SB = northern Atlantic Flyway shorebird, GU = northern Atlantic Flyway gull, PO = northern Atlantic Flyway poultry, and OB = other North American wild birds.

Through our assessment of viral dissemination among wild and domestic bird hosts sampled within the northern Atlantic Flyway and elsewhere in North America during 2015–2017, we found numerous statistically supported trends. More specifically, we found decisive support for viral dissemination from our other North American wild bird host group to northern Atlantic Flyway dabbling ducks (all gene segments), shorebirds (all internal and NA gene segments), and poultry (PB2, PA, NP, M, and NS gene segments) (Fig 3). When considering viral dissemination strictly within the northern Atlantic Flyway, we found decisive support for viral dissemination from shorebirds to gulls for all internal gene segments and the H3 gene segment (Fig 3). Also, within the northern Atlantic Flyway, we found very strong support (NP segment), strong support (PB2, PB1, NS segments), and support (PA, H5, N2 segments) for viral dissemination of specific gene segments from dabbling ducks to shorebirds (Fig 3). Strong support was found for viral dissemination of the PB1 gene segment from Atlantic Flyway dabbling ducks to poultry. We did not find BF support for dissemination of viral gene segments from gulls to any other host group within the northern Atlantic Flyway, nor from shorebirds to poultry. Topologies of phylogenies and the number of inferred Markov jumps among host groups generally corroborated the same viral dissemination trends inferred from BF support (Fig 4 and S2 File). Similarly, we found higher estimates for transitions from the other North American wild bird host group to northern Atlantic Flyway host groups as compared to strictly among northern Atlantic Flyway host groups (Fig 4 and S2 File). We also found statistical support for migration rate differences between the other North American wild bird host group and northern Atlantic Flyway host groups as compared to among northern Atlantic Flyway host groups (Fig 4 and S2 File).

In addressing sample sizes and bias, we report that the other North American wild bird host group (OB) had the largest number of gene segments for analysis in comparison to the remaining host groups (average 527 vs 65, 59, 6, and 5 segments for OB, DD, SB, GU, and PO, respectively; Table A and Fig A in S3 File). To investigate the sensitivity of this sampling bias on model results, we compared the phylogenetic model output (reconstruction of ancestral states) for each gene segment to a random prior state (tip swap) output with the expectation that if sample frequency was driving the analysis, the randomized ancestral root state probabilities would match the model results. The ancestral state reconstruction was biased toward the other North American wild bird host group, however, comparison of the model output versus random tips for multiple gene segments showed differences, indicating that the sampling bias had limited effects on our resultant models (Table B in S3 File).

## Discussion

Our study contributes a unique look at wild waterfowl migratory connectivity paired with genetic analyses to assess persistence and dissemination of influenza A viruses within the Atlantic Flyway of North America. Although important for migratory waterfowl as well as poultry production in the U.S., this flyway has been generally less sampled for wild bird-origin IAV as compared to the Pacific Flyway despite evidence for virus exchange with Europe through direct movements or shared breeding grounds in regions such as Greenland and Iceland [37,50–52]. Such virus exchange has proven ecologically and economically relevant as exemplified by frequent HPAI H5N1 detections in North America beginning in late 2021 [39,41]. Although there have been studies that provide information on virus subtypes and prevalence for waterfowl within the Atlantic Flyway [53–56], there are fewer studies that investigate viral genetic persistence or dispersal [57,58] which can help elucidate patterns in virus transport to new areas via wild birds. One exception is a long-standing IAV surveillance effort in the U.S. that focuses on shorebirds, mainly ruddy turnstones *(Arenaria interpres)*, that migrate thru Delaware Bay, coinciding with mass numbers of breeding horse-shoe crabs (*Limulus polyphemus*) [58–60]. These studies have generally provided limited evidence for IAV exchange from shorebirds to poultry, and some transfer between shorebirds and dabbling ducks [58]. Other studies that focused on dissemination of IAVs in the Atlantic Flyway have investigated waterfowl, gulls, and murres inhabiting Newfoundland, a sampling location to the north of our study sites [51,52,57,61].

We conducted our research to provide additional information on the role that dabbling ducks may contribute to virus persistence and dispersal within the Northern Atlantic Flyway. We confirmed within-season migratory connectivity for two focal waterfowl species, the mallard and American black duck, from northern breeding grounds in Maine to wintering and stopover locations in Maryland of the Chesapeake Bay. With samples obtained from these two regions, we found evidence supportive of virus persistence at the northern sites, and virus flow from breeding grounds to the more southern wintering and stopover sites of the Chesapeake Bay.

Despite banding data for waterfowl providing empirical evidence of migratory connectivity between sampling sites in Maine and Maryland, we did not find evidence of whole genome constellation dispersal of IAV by wild birds between these two states. These results are not unusual when compared to other studies [58,62] and possible, non-mutually exclusive explanations for our findings include: (1) viral dispersal of genome constellations is relatively rare and our sample was insufficient for detection of such an event, (2) Maine is likely one of multiple migratory connections (i.e. source populations of IAV) for the waterfowl sampled in Maryland, and (3) rapid accumulation of point mutations and widespread reassortment within the wild bird reservoir leads to highly transient genome constellations of IAVs. These findings of direct spatial migratory connectivity of avian hosts, lack of detection of nearly identical IAV genomes at spatially distant locations within a flyway, and evidence for dispersal of viral gene segments between northern and southern locations used by migratory birds are congruent with what has been reported for shorebirds sampled within the same flyway [58] and waterfowl sampled to the west in the Mississippi Flyway [62].

While waterfowl migratory connectivity within the flyway is to be expected [63] and aligns with previously published work on this species group [44], verifying this site-specific connection within the migratory season is important for validating assumptions of viral spread within a flyway. For instance, our finding that mallards and black ducks banded in Maine in the post-breeding season make use of the stopover or wintering habitat provided by the Chesapeake Bay within a single migratory cycle indicates that these birds could spread viruses acquired on the breeding grounds to the southern wintering grounds or migratory stopover locations. While the transmission of avian influenza viruses latitudinally within a flyway is important in the annual cycle [24,25], it can also have meaningful implications at the local level, as seasonal introduction of avian influenza viruses provides the opportunity for transmission to domestic poultry facilities that are common in this region [49]. Additionally, the congregation of waterfowl from numerous breeding grounds at this single stopover / wintering site allows for the potential spread of different viral strains across distinct breeding populations [16], both of which are important to informing IAV surveillance strategies.

Through the pairwise comparison of genome constellations of isolates recovered from dabbling ducks sampled within the northern Atlantic Flyway, we identified evidence suggestive of environmental persistence of IAVs at the northern end of the flyway. Although reports of such evidence are uncommon, two previous studies have identified highly similar IAV genome constellations between years in wild birds inhabiting Alaska [18,64]. Both laboratory and field-based evaluations provide evidence that IAVs may remain infectious in cold surface water for extended periods and potentially even overwinter when maintained at ambient environmental temperatures [20,65–67], particularly at mid to high latitudes. Therefore, additional efforts to explore whether and how the environmental persistence of IAVs may affect viral ecology within the northern Atlantic Flyway is warranted.

Our finding of statistically supported trends of viral dissemination among discrete host groups (dabbling ducks, gulls, shorebirds, poultry, and other North American wild birds) provides important insights that may be useful to ongoing and future IAV surveillance efforts within North America. For example, decisive support for viral dissemination from our other North American wild bird host group to northern Atlantic Flyway dabbling ducks, shorebirds, and poultry, elucidates the interconnected nature of the avian reservoir within which IAVs are maintained in North America. Also, within the northern Atlantic Flyway, we found support for dissemination of specific gene segments from dabbling ducks to shorebirds and poultry. Another study conducted using samples collected north of our study sites, in the Canadian portion of the Atlantic Flyway, also found evidence for frequent interspecies viral dissemination from dabbling ducks to shorebirds and poultry [57]. Virus detections and inferred patterns of dissemination among host groups may be influenced by numerous factors including geographic distribution of sampling, sampling efforts, availability of complementary reference data (i.e., virus sequences) available in public databases, ecological factors related to bird movements and connectivity on the landscape [18,68], and host immunity at the time of sampling [69–71]. Taking into account these complexities, continued surveillance across these groups is important to understanding virus movement within and across wild populations as well as at the interface between wild birds and poultry.

Our finding of decisive support for viral dissemination from northern Atlantic Flyway shorebirds to gulls provides evidence to further support that spillover of IAVs isolated from ruddy turnstones, a common viral host in the eastern United States and throughout the Neotropics [58,72–75], contributes to the annual epidemic historically observed each spring at Delaware Bay [76], which is located just north of the Chesapeake Bay. In turn, the lack of statistical support for viral dissemination from either shorebirds or gulls to Atlantic Flyway poultry over the course of our study suggests this annual epidemic may not lead to frequent detections of viral spillover in local domestic birds. It should be noted, however, that two prior reports have indicated that H7N3 viruses isolated from ruddy turnstones from Delaware Bay shared common ancestry with viruses affecting poultry in the Canadian provinces of British Columbia in 2004 [77] and Saskatchewan in 2007 [78]. Additional surveillance may help elucidate whether these connections reflect virus movements between these populations or are due to lack of sequences from intermediate hosts.

As noted previously, availability of sequences for genetic analyses in public databases may be biased spatially, temporally, and taxonomically. We recognize these biases within our analyses, with the other North American Wild Bird host group (OB) having a disproportionately large number of samples compared to the defined Atlantic Flyway host groups (Table A and Fig A in S3 File). A common approach to addressing sampling frequency bias is to down sample, or reduce the number of inputs within the larger groups, which is often done with very large datasets [25,79]. We chose not to subsample as our dataset contained limited information for various host groups and spanned only three years. Thus, we were concerned that down sampling would introduce other biases and/or preclude statistical rigor for deriving inference. Therefore, we instead opted to investigate the potential effects of biases in our analyses. When we compared our main model outputs with a random tip swap analysis, the sensitivity analysis provided evidence that inferred trends in viral dissemination were driving the ancestral reconstruction and that sampling frequency of host groups had only limited impacts on the model results. As such, while we acknowledge that our input data has inherent sampling biases, we also believe that our results provide useful inference on general trends of viral dissemination within the Atlantic Flyway.

Understanding the diversity of viruses circulating among populations is important for identifying potential risks to domestic and wild bird health. A broad diversity of IAVs was detected in this study, with surface proteins including hemagglutinin H1–4, H6, H7–11 and neuraminidase N1–4, N6, and N9. The findings of H4N6 as subtype IAVs in ducks sampled in Maine and Maryland in each year is consistent with reports of IAVs of this subtype as being common within dabbling ducks sampled at both northern and more southern areas of North America [15,17,80–84]. Additionally, the isolation of H3N8 and H3N2 IAVs in ducks sampled in Maine and H1N1 IAVs in birds sampled in Maryland in each sampling year is congruent with previous contemporary detections of viruses of these subtypes in dabbling ducks sampled elsewhere in northern North America and at lower latitudes within the United States, respectively [17,81–83].

Similar to studies conducted in other flyways [8,22,23], we observed evidence for higher prevalence of IAVs among wild birds sampled in the northern sites in Maine than in Maryland, and the contrary relationship when considering antibody seroprevalence suggesting that the majority of infections occur when immunologically naïve juveniles enter the population (i.e., prior to beginning fall migration) and decrease as the population builds immunity prior to arrival at sites along migration such as the Chesapeake Bay. Prevalence estimates detected in this study (32% and 15% M+ in Maine and Maryland, respectively) fall in similar range to those reported in previous works in the Atlantic Flyway when accounting for species and latitude. Groepper et al. [85], reported a range of prevalence from 15.1 to 21.2% in the Atlantic Flyway from 2007–2009, similar to our Maryland samples, which are located mid-latitude across the larger flyway. A 2017 study within the Delmarva Peninsula reported average prevalence of 14% across all dabbling ducks, with species values of 28% for black ducks and 10% for mallards [53]. One study which reported a lower prevalence of 3.1 and 1.5% for black ducks and mallards, respectively, [55] sampled over a wider time span within the annual cycle (September through March as opposed to September thru December for the other two studies) with half the samples collected in March, and showed reduced prevalence as the post-breeding season progressed.

## Conclusions

This study provides a unique assessment of linkages between bird movements and IAV dissemination patterns with a flyway perspective and has important global implications given connectivity between the northern Atlantic Flyway and eastern Canada, other parts of the U.S., Central and South America, as well as across the Atlantic Ocean to Europe via Greenland and Iceland. Congruent with previous broad-scale studies, prevalence of IAVs was inferred to be higher in the breeding season’s northern latitudes and seroprevalence and mixed infections were higher southward in the flyway where waterfowl stop over during migration and intermix throughout the wintering season with birds that had bred elsewhere. Evidence for potential environmental persistence of virus was observed at the northern latitudes within the flyway, and signs of viral gene flow was evident between sampling areas sharing migratory connectivity. Several patterns of viral dissemination were supported across host groups within the northern half of the flyway (Maine to the Chesapeake Bay, Maryland) including gene flow from dabbling ducks to shorebirds and poultry, which have implications to understanding virus movement across wild populations as well as into the domestic poultry system. Additional efforts to evaluate avian influenza prevalence and persistence across other times of the year, such as from winter through spring migration would be valuable next steps. This work provides an important contribution to understanding IAV in waterfowl in the northern portion of the Atlantic Flyway and builds grounds for development of future work in this region, particularly as HPAI has now been detected in wild birds within the Atlantic Flyway and has subsequently spread to other flyways within North America.

## Materials and methods

### Ethics statement

All handling of birds was performed by trained personnel. The study was approved by the USGS Eastern Ecological Center Animal Care and Use Committee (2014-02P). Live-captured waterfowl were banded and sampled under banding permit 23913 from the U.S. National Bird Banding Laboratory.

### Waterfowl band recovery (migratory connectivity)

To assess migratory connectivity along the Northern Atlantic Flyway (Maine to Chesapeake Bay, Maryland, which includes the following contiguous states within U.S.: Connecticut, Delaware, Maine, Maryland, Massachusetts, New Hampshire, New Jersey, New York, Pennsylvania, Rhode Island, Vermont, Virginia, West Virginia) we requested all mallard and black duck encounter (reported by the general public, primarily hunter harvest data) and recapture (reported by licensed banders primarily from targeted capture for banding) data from the North American Bird Banding Program via the BBL. These species were selected for their importance to waterfowl populations in the Atlantic Flyway, their importance as wild reservoirs for avian influenza viruses [86,87], and their suspected high connectivity between Maine and the Chesapeake Bay [88]. The acquired data were filtered to include only birds banded in Maine over the past decade (2011–2021) and encountered or recaptured within the same migratory cycle in which they were initially banded (e.g., banded on breeding grounds in Maine and encountered in the fall or winter which spans until February of the following calendar year). We manually summed the number of birds encountered or recaptured within the Maryland and Virginia portions of the Chesapeake Bay Watershed following the processing steps described above. Migratory connections were visualized in ArcMap 10.4 [89].

### Field sampling

We sampled both hunter harvested and live captured dabbling ducks from August to December of 2015, 2016, and 2017. Hunter-harvested birds were collected opportunistically from bird processing facilities across the Eastern Shore of Maryland (a portion of the Chesapeake Bay watershed known for abundant waterfowl) where hunters bring harvested birds for cleaning. One cloacal and one oropharyngeal swab [90] were collected from each bird using sterile polyester-tipped applicators and placed as paired samples (by bird) in vials containing 2 mL of cold virus transport medium (VTM) supplemented with antimicrobials including 2 ml of Brain Heart Infusion media (Becton Dickinson and Co., Sparks, MD) supplemented with penicillin G (1,000 units/ml), streptomycin (1 mg/ml), kanamycin (0.5 mg/ml), gentamicin (0.25 mg/ml), and amphotericin B (0.025 mg/ml) (Sigma Chemical Company, St. Louis, MO). Swabs were stored on ice for <12 hours during sample collection and transport to a holding facility where they were then stored at -80°C until being shipped on dry ice to the laboratory where they were held at -80°C until processing. Live captured birds, caught via swim-in traps baited with corn, were sampled in Maine at Christina Reservoir (46.693, -76.891) and Lake Josephine (46.673, -67.904) and surrounding wetlands, at Moosehorn National Wildlife Refuge (45.056, -67.297), and in the Maryland portion of the Chesapeake Bay at Poplar Island; 38.762, -76.383). Capture efforts targeted mallards and black ducks, although other dabbling duck species were sampled opportunistically. Upon capture, birds were placed in large holding containers and stored out of direct sunlight until they could be individually processed and released at the capture location. Each bird was marked with a metal U.S. Geological Survey leg band. All live-captured birds were swabbed as described above for hunter harvested birds. Additionally, up to 3 mL of blood was taken from the jugular or leg vein of live-captured birds and placed in a BD Vacutainer SST tube (BD, Franklin Lakes, New Jersey, USA) where it was inverted 3–5 times to aid homogenization and placed on ice. Upon return to the lab, blood samples were spun in a centrifuge for 20 minutes at 2500 rpm to separate serum and red blood cells. Serum samples were then poured into individual vials and stored at -80°C until shipped on dry ice for further processing. All capture, handling, and marking procedures were conducted in accordance with the methodology outlined by the Food and Agriculture Organization of the United Nations [91].

### Antibody testing

We tested serum samples by commercial blocking enzyme-linked immunosorbent assay (bELISA, IDEXX Laboratories, Westbrook, ME) for influenza A virus antibodies as described by the manufacturer. An initial serum-to-negative control (S/N) absorbance ratio < 0.5 represents the cutoff threshold recommended by the manufacturer; however, this threshold is most sensitive to high antibody titers that occur with primary immune response from recent infections. Multiple studies show that S/N cutoff threshold of < 0.7 increases sensitivity while preserving specificity in wild waterfowl that may have been exposed weeks to months prior to sampling [92–94]. For this reason, and to allow for comparison across studies in the literature, we provide classifications using both thresholds. We used a generalized linear model with a logit link (~ Year + Flyway Location + Species) to assess trends in seroprevalence between the areas sampled in this study, with post-hoc pairwise comparisons of species performed via the “emmeans” function in R [89,95]. This model was run for seroprevalence at both the S/N < 0.5 and 0.7 thresholds.

### Influenza A virus screening

We extracted viral RNA from all swab samples using the MagMAX-96 AI/ND Viral RNA Isolation Kit (Ambion/Applied Biosystems, Foster City, CA) and screened using real-time reverse transcriptase-PCR (rRT-PCR) that targeted the IAV matrix (M) gene [96]. Any sample that did not yield a cycle threshold (Ct) value ≤45 was considered negative for IAV [96]. We attempted virus isolation on all M positive (M+) samples by inoculating a total 1mL of VTM into the allantoic cavities of three 9-11-day-old embryonated chicken eggs [97] and incubating at 37°C for 120 hours. Amnioallantoic fluid was collected and tested by hemagglutination assay [98]. We extracted viral RNA from all hemagglutinating samples using the QIAamp Viral RNA mini kit (Qiagen, Inc., Germantown, MD) and IAV were identified by RT-PCR targeting the M gene [96,99]. We used generalized linear models (~ Year + Flyway Location + Species) to assess trends in both influenza A virus prevalence (matrix gene, M+) and virus isolation ratios (VI/M+) between the areas sampled in this study, with post-hoc pairwise comparisons of species performed via the “emmeans” function in R [89,95].

### RNA extraction and genetic sequencing

RNA was extracted from amnioallantoic egg fluids for all putative virus isolation-positive samples using the QIAamp viral RNA mini kit (Qiagen Inc.; Germantown, MD, USA). Complementary DNA was synthesized and subsequently amplified, visualized, and purified per previously reported methods [81]. Genomic sequences for IAVs were obtained by using Nextera XT DNA library preparation kits (Illumina, Inc.; San Diego, CA, USA), pooling indexed libraries, and sequencing on an Illumina MiSeq using either 500 or 600-cycle reagent kits with paired-end reads. Reads were assembled using a customized workflow on Geneious R11.0.4 (Biomatters Ltd.; Auckland, New Zealand) using reference data for influenza A viruses obtained from GenBank [100]. GenBank accession numbers for genomic sequence data for isolates generated and sequenced for this study are: MT 420910–MT421775.

### Assessment of viral environmental persistence and viral dispersal

To assess (a) genetic evidence for possible environmental persistence of IAVs at study sites in Maine or Maryland or (b) viral dispersal between sites, we calculated shared pairwise nucleotide identity (a measure of genetic similarity) among viral genomes isolated from wild bird samples collected from Maine and Maryland during 2015–2017. More specifically, we calculated nucleotide identity of each of eight gene segments among all complete, non-mixed infection genomes sequenced as part of this study (n = 93), as well as full non-mixed infection genomes for reference IAVs from wild birds reported on GenBank (accessed 2 June 2020) from Maine during 2015–2017 and Maryland during 2015–2016 (n = 9).

Regarding viral persistence, we considered isolates identified from the same state but different years that shared > 99% nucleotide identity at all eight gene segments to be suggestive of persistence between years. If less than eight gene segments shared >99% nucleotide identity, we did not consider isolates to be indicative of persistence; rather, we interpreted such findings as suggestive of genetic shift and/or genetic drift of genome constellations within wild bird reservoir hosts. We considered a lack of gene segments sharing > 99% nucleotide identity among isolates derived from samples from the same state but different years to be suggestive of the extirpation of viral genome constellations between years.

Regarding viral dispersal, isolates from birds sampled in different states that shared >99% nucleotide identity at all eight gene segments were considered to be indicative of viral dispersal between sites. If at least one but less than eight gene segments shared >99% identity, we considered this to be indicative of viral gene flow between sites in conjunction with genetic shift and/or drift of genome constellations maintained by wild bird reservoir hosts. If no gene segments shared > 99% identity we considered this to be suggestive of infrequent or no viral gene flow among populations of birds sampled in Maine and Maryland.

We expected some isolates recovered from samples collected in the same state and the same year to share > 99% nucleotide identity at all eight gene segments on account of local viral transmission within the wild bird reservoir. Thus, we did not test any hypotheses using such comparisons, though they were identified and counted.

### Assessment of viral dissemination among diverse avian hosts

To assess support for viral dissemination among wild and domestic bird hosts sampled within the northern Atlantic Flyway and wild birds sampled concurrently elsewhere in North America during 2015–2017, we conducted comparative phylogenetic analyses using discrete host groups. To do so, we first generated an input file for each IAV internal gene segment (PB2, PB1, PA, NP, M, and NS) as well as for the hemagglutinin H1, H3, and H5 and neuraminidase N1, N2, and N8 gene segments. Each input file was populated with complete coding sequences for gene segments of isolates generated in this study and reference complete coding sequences for gene segments of avian-origin IAVs from samples collected elsewhere from North America during 2015–2017 as reported on GenBank (accessed 26 June 2020). Each sequence was assigned one of the following five discrete host groups: northern Atlantic Flyway dabbling duck (DD), northern Atlantic Flyway shorebird (SB), northern Atlantic Flyway gull (GU), northern Atlantic Flyway poultry (PO), or other North American wild bird (OB). For identical sequences, we included data for only a single representative virus for each host group. We also removed sequences from the other North American wild bird host groups that clearly represented domestic bird origin sequences and those for which wild status could not be inferred (i.e., duck, chicken, turkey, goose, Muscovy, Guinea fowl, peafowl, spot-billed duck, pheasant, and quail). Sequence information from the few IAV sequences originating from the southern Atlantic Flyway (n = 10; from North Carolina, South Carolina, and Florida) and from hosts other than dabbling ducks, shorebirds, gulls, and poultry from the northern Atlantic Flyway (n = 5; from geese and sea ducks) were included in our “other” North American wild bird host group. Resultant input files contained at least 127 (HA H1gene segment) and up to 1306 (PA gene segment) sequences per gene segment, with largest samples sizes for internal gene segments (n ≥ 1012).

A preliminary maximum likelihood phylogenetic tree was next generated for each gene segment using RAxML v8.2.12 [101] to identify the phylogenetic outlier sequences which were subsequently removed from the dataset to facilitate downstream model convergence. Trees were generated using a general time reversible nucleotide substitution model with gamma distribution of rates. The clock signal was investigated for each maximum likelihood phylogenetic tree using Tempest v1.5 [102]. Viral sequences with clock rates substantially lower or higher than the mean rate (lower 2.5 percentile) were classified as outliers.

We next conducted comparative Bayesian phylogenetic analyses for each gene segment using BEAST v.1.10.4 [103] incorporating an uncorrelated lognormal relaxed molecular clock that allows for rate variation across lineages. The SRD06 codon position model [104] was used along with a constant coalescent tree prior [105]. A minimum of four independent runs of 150 million generations were performed and combined after removal of burn-in to achieve an Effective Sample Size of >200 as diagnosed in Tracer v1.6 [106]. Empirical tree sets were constructed by combining the last 1000 trees of each run for subsequent discrete traits analysis.

We next applied discrete trait diffusion models to estimate the rate of transitions among the five host groups described above: DD, SB, GU, PO, OB (the first four being northern Atlantic Flyway dabbling ducks, shorebirds, gulls, and poultry and the 5^th^ being other birds). We used a non-reversible continuous-time Markov chain model to estimate the migration rates between different host groups. We performed Bayesian stochastic search variable selection (BSSVS) to reduce the complexity of the models and to identify significantly non-zero migration rates using a binary indicator (I). Based on I, Bayes factors (BF) were calculated using SPREAD v1.0 [107]. Rates were considered statistically supported using BF as follows: weak/poor support when BF < 3, support when 3 ≤ BF < 10, strong support when 10 ≤ BF < 100, very strong support when 100 ≤ BF < 1000, decisive support when BF ≥ 1000.

Additionally, we assessed statistical support for differences of migration rate between northern Atlantic Flyway host groups and the other North American wild bird hosts by computing and comparing BF. We estimated the BF for differences in migration rates (r) by the ratio of posterior odds (P(r1 > r2 | Data)/P(r2 > r1 | Data)) versus prior odds P(r1 > r2)/P(r2 > r1), where the prior odds ratio was approximately set to 1 [108]. To assess the contribution of each host group as a viral source or sink in the migration network, state jumps at the tree nodes, representing an inferred state transition event, were recorded. We used a non-reversible model and therefore the direction of gene flow between states was used to assign hosts groups as either source or sink for each transition. We generated heat maps to represent the average number of jumps per year estimated from the last 3000 posterior sampled trees. Only the Markov jumps between 2015 and 2017 were analyzed to be consistent with the sampling period for sites in Maine and Maryland and the year of sampling for viral sequences obtained from GenBank. Markov jumps were extracted from the complete jump history using a custom R script [109] to visualize heat maps.

No down sampling was applied to genetic segments for the Bayesian phylogenetic analyses as the overall sample size was manageable (segments were obtained across only 3 years of data) and we aimed to preserve the genetic diversity within each discrete host category. To assess potential sampling frequency bias across host states on the model results, we performed a sensitivity analysis comparing observed models versus randomized apriori models. More specifically, we compared the probability of the estimated ancestral root state from each model (model output) with the probability of an ancestral root state obtained when the discrete host groups were randomized at the model tips (“tip swap”) during the Markov process both with and without BSSVS analysis [110,111]. With this process, we expected that differing relative proportions per host group in the randomized tip swap would reflect differences in sample size. Then, if the probabilities in the model output were to match these patterns, we would assume that sampling bias may have influenced the model output; however, if proportions of the host tip assignments were dissimilar between the model output and randomized analyses, there is indication that trends in viral dissemination, not strictly sampling bias were driving the output. Further detail on this process is described in S3 File).

## Supporting information

S1 Table Swab and serum samples per site, capture type, and species. The number of paired cloacal and oropharyngeal swabs and blood serum samples collected (C) from dabbling duck species and analyzed (A) to test for influenza A virus in Maine (ME) and Maryland (MD) by year, species, and capture method from 2015–2017. Live-captured birds (LC), caught via swim-in traps baited with corn, were sampled in both Maine and Maryland, while hunter harvested birds (HH) were sampled only in Maryland. Waterfowl species are identified via their alpha codes as follows: ABDU (American Black Duck), ABDU x MALL (American Black Duck x Mallard Hybrid), AGWT (American Green-winged Teal), BWTE (Blue-winged Teal), GADW (Gadwall), MALL (Mallard), NOPI (Northern Pintail), and NOSH (Northern Shoveler).
(DOCX)

S2 Table Influenza A virus prevalence, serology, and subtyping from dabbling duck samples. The prevalence of influenza A virus as identified via rRT-PCR and virus isolation (VI), avian influenza antibodies as identified via bELISA, and the genetic sequencing results for wild dabbling ducks sampled in Maine (ME) and Maryland (MD) from 2015–2017. Waterfowl species are identified via their alpha codes as follows: ABDU (American Black Duck), ABDU x MALL (American Black Duck x Mallard Hybrid), AGWT (American Green-winged Teal), BWTE (Blue-winged Teal), GADW (Gadwall), MALL (Mallard), NOPI (Northern Pintail), and NOSH (Northern Shoveler).
(DOCX)

S1 File The percentage similarity between avian influenza virus isolates at each of eight gene segments. Tab 1: The number of gene segments that are >99% similar between two influenza A virus isolates. Tabs 2 through 9: Percent similarity between gene segments for PB2, PB1, PA, HA, NP, NA, M, and NS gene segments.
(XLSX)

S2 File Phylogenetic trees and Markov jumps for all gene segments of isolated influenza A viruses. Bayesian phylogenetic tree (Panel A), heat map inferred number of Markov jumps among hosts groups (Panel B), plot of estimates for transitions from the other North American wild bird host group to northern Atlantic Flyway host groups as compared to strictly among northern Atlantic Flyway host groups (Panel C), and plot of ratios of posterior odds versus prior odds to infer differences in migration rate estimates between the other North American wild bird host group and northern Atlantic Flyway host groups as compared to strictly among northern Atlantic Flyway host groups (Panel D) for the PB1, PA, NP, M, and NS internal gene segments as well as hemagglutinin H3, and H5 and neuraminidase N2, and N8 gene segments. Note that for gene segments hemagglutinin H1 and neuraminidase N1, support for transmission within the Atlantic Flyway hosts was not determined (BF less than 3) and hence, panels C and D are not included.
(PDF)

S3 File Model sensitivity to sampling bias. This file lists the number of sequences per discrete host group and describes the sampling bias sensitivity analysis applied to explore whether the uneven sample sizes among host groups (particularly Other Birds; OB) greatly affected the phylogenetic analysis model results. Table A: List of segments for phylogenetic analysis per discrete host group. Table B: Tip-state randomization to examine ancestral root state probabilities. Fig A: Bar graph showing number of segments for phylogenetic analysis per discrete host group.
(DOCX)

S4 File Printed phylogenetic trees with tip labels and host group information. This file contains phylogenetic trees printed and attached in a PDF format for those who do not have FigTree or other software to view the treeplots.
(PDF)

S5 File Treeplot format phylogenetic trees with tip labels and host group information. This file contains Treeplot format phylogenetic trees which can be visualized and manipulated using FigTree software available at: evomics.org.
(EXE)
