# Supplementary material for: Maintenance and dissemination of avian-origin influenza A virus within the northern Atlantic Flyway of North America
Source: PLoS Pathog. 2022 Jun 6;18(6):e1010605. doi: 10.1371/journal.ppat.1010605 (PMC9203021; doi:10.1371/journal.ppat.1010605)
Supplement: S1 Table — The number of paired cloacal and oropharyngeal swabs and blood serum samples collected (C) from dabbling duck species and analyzed (A) to test for influenza A virus in Maine (ME) and Maryland (MD) by year, species, and capture method from 2015–2017. Live-captured birds (LC), caught via swim-in traps baited with corn, were sampled in both Maine and Maryland, while hunter harvested birds (HH) were sampled only in Maryland. Waterfowl species are identified via their alpha codes as follows: ABDU (American Black Duck), ABDU x MALL (American Black Duck x Mallard Hybrid), AGWT (American Green-winged Teal), BWTE (Blue-winged Teal), GADW (Gadwall), MALL (Mallard), NOPI (Northern Pintail), and NOSH (Northern Shoveler). (DOCX) [file ppat.1010605.s001.docx]

Table S1. The number of paired cloacal and oropharyngeal swabs and blood serum samples collected (C) from dabbling duck species and analyzed (A) to test for influenza A virus in Maine (ME) and Maryland (MD) by year, species, and capture method from 2015 – 2017. Live-captured birds (LC), caught via swim-in traps baited with corn, were sampled in both Maine and Maryland, while hunter harvested birds (HH) were sampled only in Maryland. Waterfowl species are identified via their alpha codes as follows: ABDU (American Black Duck), ABDU x MALL (American Black Duck x Mallard Hybrid), AGWT (American Green-winged Teal), BWTE (Blue-winged Teal), GADW (Gadwall), MALL (Mallard), NOPI (Northern Pintail), and NOSH (Northern Shoveler).

|  |  |  | 2015 | | | | |  | 2016 | | | | |  | 2017 | | | | |  | Total | | | | |
| --- | --- | --- | --- | --- | --- | --- | --- | --- | --- | --- | --- | --- | --- | --- | --- | --- | --- | --- | --- | --- | --- | --- | --- | --- | --- |
|  |  |  | Swabs | |  | Serum | |  | Swabs | |  | Serum | |  | Swabs | |  | Serum | |  | Swabs | |  | Serum | |
| State | Capture Type | Species | C | A |  | C | A |  | C | A |  | C | A |  | C | A |  | C | A |  | C | A |  | C | A |
| ME | LC | ABDU | 64 | 64 |  | 0 | 0 |  | 103 | 103 |  | 0 | 0 |  | 98 | 98 |  | 0 | 0 |  | 265 | 265 |  | 0 | 0 |
|  |  | ABDU x MALL | 2 | 2 |  | 0 | 0 |  | 3 | 3 |  | 0 | 0 |  | 2 | 2 |  | 0 | 0 |  | 7 | 7 |  | 0 | 0 |
|  |  | BWTE | 3 | 3 |  | 3 | 0 |  | 0 | 0 |  | 0 | 0 |  | 0 | 0 |  | 0 | 0 |  | 3 | 3 |  | 3 | 0 |
|  |  | MALL | 151 | 151 |  | 137 | 126 |  | 160 | 160 |  | 116 | 116 |  | 176 | 176 |  | 0 | 0 |  | 487 | 487 |  | 253 | 242 |
| MD | HH | ABDU | 4 | 4 |  | 0 | 0 |  | 0 | 0 |  | 0 | 0 |  | 0 | 0 |  | 0 | 0 |  | 4 | 4 |  | 0 | 0 |
|  |  | AGWT | 45 | 45 |  | 0 | 0 |  | 29 | 29 |  | 0 | 0 |  | 0 | 0 |  | 0 | 0 |  | 74 | 74 |  | 0 | 0 |
|  |  | BWTE | 7 | 7 |  | 0 | 0 |  | 0 | 0 |  | 0 | 0 |  | 0 | 0 |  | 0 | 0 |  | 7 | 7 |  | 0 | 0 |
|  |  | GADW | 0 | 0 |  | 0 | 0 |  | 2 | 2 |  | 0 | 0 |  | 0 | 0 |  | 0 | 0 |  | 2 | 2 |  | 0 | 0 |
|  |  | MALL | 18 | 18 |  | 0 | 0 |  | 24 | 24 |  | 0 | 0 |  | 0 | 0 |  | 0 | 0 |  | 42 | 42 |  | 0 | 0 |
|  |  | NOPI | 5 | 5 |  | 0 | 0 |  | 10 | 10 |  | 0 | 0 |  | 0 | 0 |  | 0 | 0 |  | 15 | 15 |  | 0 | 0 |
|  |  | NOSH | 0 | 0 |  | 0 | 0 |  | 11 | 11 |  | 0 | 0 |  | 0 | 0 |  | 0 | 0 |  | 11 | 11 |  | 0 | 0 |
| MD | LC | ABDU | 156 | 156 |  | 150 | 150 |  | 102 | 102 |  | 86 | 85 |  | 0 | 0 |  | 0 | 0 |  | 258 | 258 |  | 236 | 235 |
|  |  | ABDU x MALL | 16 | 16 |  | 16 | 16 |  | 9 | 9 |  | 6 | 6 |  | 0 | 0 |  | 0 | 0 |  | 25 | 25 |  | 22 | 22 |
|  |  | AGWT | 2 | 2 |  | 2 | 2 |  | 0 | 0 |  | 0 | 0 |  | 0 | 0 |  | 0 | 0 |  | 2 | 2 |  | 2 | 2 |
|  |  | BWTE | 1 | 1 |  | 0 | 0 |  | 0 | 0 |  | 0 | 0 |  | 0 | 0 |  | 0 | 0 |  | 1 | 1 |  | 0 | 0 |
|  |  | MALL | 267 | 267 |  | 256 | 256 |  | 291 | 291 |  | 215 | 214 |  | 0 | 0 |  | 0 | 0 |  | 558 | 558 |  | 471 | 470 |
|  |  | NOPI | 57 | 57 |  | 57 | 57 |  | 3 | 3 |  | 3 | 3 |  | 0 | 0 |  | 0 | 0 |  | 60 | 60 |  | 60 | 60 |
| Total |  |  | 798 | 798 |  | 621 | 607 |  | 747 | 747 |  | 426 | 424 |  | 276 | 276 |  | 0 | 0 |  | 1821 | 1821 |  | 1047 | 1031 |
