# Supplementary material for: Maintenance and dissemination of avian-origin influenza A virus within the northern Atlantic Flyway of North America
Source: PLoS Pathog. 2022 Jun 6;18(6):e1010605. doi: 10.1371/journal.ppat.1010605 (PMC9203021; doi:10.1371/journal.ppat.1010605)
Supplement: S2 Table — The prevalence of influenza A virus as identified via rRT-PCR and virus isolation (VI), avian influenza antibodies as identified via bELISA, and the genetic sequencing results for wild dabbling ducks sampled in Maine (ME) and Maryland (MD) from 2015–2017. Waterfowl species are identified via their alpha codes as follows: ABDU (American Black Duck), ABDU x MALL (American Black Duck x Mallard Hybrid), AGWT (American Green-winged Teal), BWTE (Blue-winged Teal), GADW (Gadwall), MALL (Mallard), NOPI (Northern Pintail), and NOSH (Northern Shoveler). (DOCX) [file ppat.1010605.s002.docx]

S2 Table. The prevalence of influenza A virus as identified via rRT-PCR and virus isolation (VI), avian influenza antibodies as identified via bELISA, and the genetic sequencing results for wild dabbling ducks sampled in Maine (ME) and Maryland (MD) from 2015 – 2017. Waterfowl species are identified via their alpha codes as follows: ABDU (American Black Duck), ABDU x MALL (American Black Duck x Mallard Hybrid), AGWT (American Green-winged Teal), BWTE (Blue-winged Teal), GADW (Gadwall), MALL (Mallard), NOPI (Northern Pintail), and NOSH (Northern Shoveler). Dashes indicate instances where no serology data was collected.

|  |  |  | rRT-PCR+VI | | | | |  | Serology | | | | | |  | Subtyping |
| --- | --- | --- | --- | --- | --- | --- | --- | --- | --- | --- | --- | --- | --- | --- | --- | --- |
|  |  |  |  | Ct <= 45 | |  |  |  |  | S/N < .5 | |  | S/N < .7 | |  |  |
| State | Year | Species | n | M+ | M+/n | VI+ | VI+/n |  | n | ELISA+ | ELISA+/n |  | ELISA+ | ELISA+/n |  | Subtypes (count) |
| ME | 2015 | Total | 220 | 44 | 20.0% | 16 | 7.3% |  | 126 | 53 | 42.1% |  | 87 | 69.0% |  |  |
|  |  | ABDU | 64 | 15 | 23.4% | 2 | 3.1% |  | - | - | - |  | - | - |  | H3N2 (2) |
|  |  | ABDU x MALL | 2 | 0 | 0.0% | 0 | 0.0% |  | - | - | - |  | - | - |  |  |
|  |  | BWTE | 3 | 3 | 100.0% | 3 | 100.0% |  | - | - | - |  | - | - |  | H4N6 (3) |
|  |  | MALL | 151 | 26 | 17.2% | 11 | 7.3% |  | 126 | 53 | 42.1% |  | 87 | 69.0% |  | H3N2 (6); H3N8 (3); H4N6 (1); H10N3 (1) |
| ME | 2016 | Total | 266 | 128 | 48.1% | 30 | 11.3% |  | 116 | 20 | 17.2% |  | 37 | 31.9% |  |  |
|  |  | ABDU | 103 | 60 | 58.3% | 14 | 13.6% |  | - | - | - |  | - | - |  | H3N8 (2); H4N6 (8); H9N9 (2) mixed (1[H9 N6+N9], 1[H4 N6+N9]) |
|  |  | ABDU x MALL | 3 | 3 | 100.0% | 0 | 0.0% |  | - | - | - |  | - | - |  |  |
|  |  | MALL | 160 | 65 | 40.6% | 16 | 10.0% |  | 116 | 20 | 17.2% |  | 37 | 31.9% |  | H3N2(1); H3N8 (3); H4N6 (10); H11N1 (1); mixed (1[H4 N6]) |
| ME | 2017 | Total | 276 | 75 | 27.2% | 34 | 12.3% |  | - | - | - |  | - | - |  |  |
|  |  | ABDU | 98 | 23 | 23.5% | 6 | 6.1% |  | - | - | - |  | - | - |  | H3N8 (1); H4N6 (2); H9N2 (2); mixed (1[H3+H4 N6+N8]) |
|  |  | ABDU x MALL | 2 | 1 | 50.0% | 1 | 50.0% |  | - | - | - |  | - | - |  | H4N6 (1) |
|  |  | MALL | 176 | 51 | 29.0% | 27 | 15.3% |  | - | - | - |  | - | - |  | H3N2 (1); H3N6 (2); H3N8 (5); H4N6 (7); H8N4 (4); H9N2 (1); mixed (5[H3+H4 N6+N8], 1(H1+H3+H3 N1+N8+N8), 1[H3+H3 N8+N8]) |
| MD | 2015 | Total | 578 | 82 | 14.2% | 14 | 2.4% |  | 481 | 237 | 49.3% |  | 351 | 73.0% |  |  |
|  |  | ABDU | 160 | 19 | 11.9% | 3 | 1.9% |  | 150 | 82 | 54.7% |  | 110 | 73.3% |  | H1N1 (1); H4N6 (1); H4N9 (1) |
|  |  | ABDU x MALL | 16 | 2 | 12.5% | 1 | 6.3% |  | 16 | 7 | 43.8% |  | 11 | 68.8% |  | H11N2 (1) |
|  |  | AGWT | 47 | 1 | 2.1% | 0 | 0.0% |  | 2 | 0 | 0.0% |  | 0 | 0.0% |  |  |
|  |  | BWTE | 8 | 0 | 0.0% | 0 | 0.0% |  | - | - | - |  | - | - |  |  |
|  |  | MALL | 285 | 52 | 18.2% | 10 | 3.5% |  | 256 | 127 | 49.6% |  | 198 | 77.3% |  | H3N1 (1); H4N6 (3); H4N9 (1); H6N2 (2); H11N2 (2); mixed (1[H4 N2+N9]) |
|  |  | NOPI | 62 | 8 | 12.9% | 0 | 0.0% |  | 57 | 21 | 36.8% |  | 32 | 56.1% |  |  |
| MD | 2016 | Total | 481 | 74 | 15.4% | 15 | 3.1% |  | 308 | 172 | 55.8% |  | 232 | 75.3% |  |  |
|  |  | ABDU | 102 | 20 | 19.6% | 2 | 2.0% |  | 85 | 47 | 55.3% |  | 67 | 78.8% |  | H1N1 (2) |
|  |  | ABDU x MALL | 9 | 0 | 0.0% | 0 | 0.0% |  | 6 | 2 | 33.3% |  | 4 | 66.7% |  |  |
|  |  | AGWT | 29 | 2 | 6.9% | 1 | 3.4% |  | - | - | - |  | - | - |  | H4N6 (1) |
|  |  | GADW | 2 | 0 | 0.0% | 0 | 0.0% |  | - | - | - |  | - | - |  |  |
|  |  | MALL | 315 | 49 | 15.6% | 11 | 3.5% |  | 214 | 122 | 57.0% |  | 158 | 73.8% |  | H1N2 (1); H3N6 (5); H11N9 (1); mixed (2[H1 N1+N2], 1[H3 N1+N2+N6], 1[H1+H5 N2]) |
|  |  | NOPI | 13 | 2 | 15.4% | 0 | 0.0% |  | 3 | 1 | 33.3% |  | 3 | 100.0% |  |  |
|  |  | NOSH | 11 | 1 | 9.1% | 1 | 9.1% |  | - | - | - |  | - | - |  | H11N9 (1) |
