# Supplementary material for: Maintenance and dissemination of avian-origin influenza A virus within the northern Atlantic Flyway of North America
Source: PLoS Pathog. 2022 Jun 6;18(6):e1010605. doi: 10.1371/journal.ppat.1010605 (PMC9203021; doi:10.1371/journal.ppat.1010605)

Bayesian phylogenetic tree (Panel A), heat map inferred number of Markov jumps among hosts groups (Panel B), plot of estimates for transitions from the other North American wild bird host group to northern Atlantic Flyway host groups as compared to strictly among northern Atlantic Flyway host groups (Panel C), and plot of ratios of posterior odds versus prior odds to infer differences in migration rate estimates between the other North American wild bird host group and northern Atlantic Flyway host groups as compared to strictly among northern Atlantic Flyway host groups (Panel D) for the PB1 gene segment.

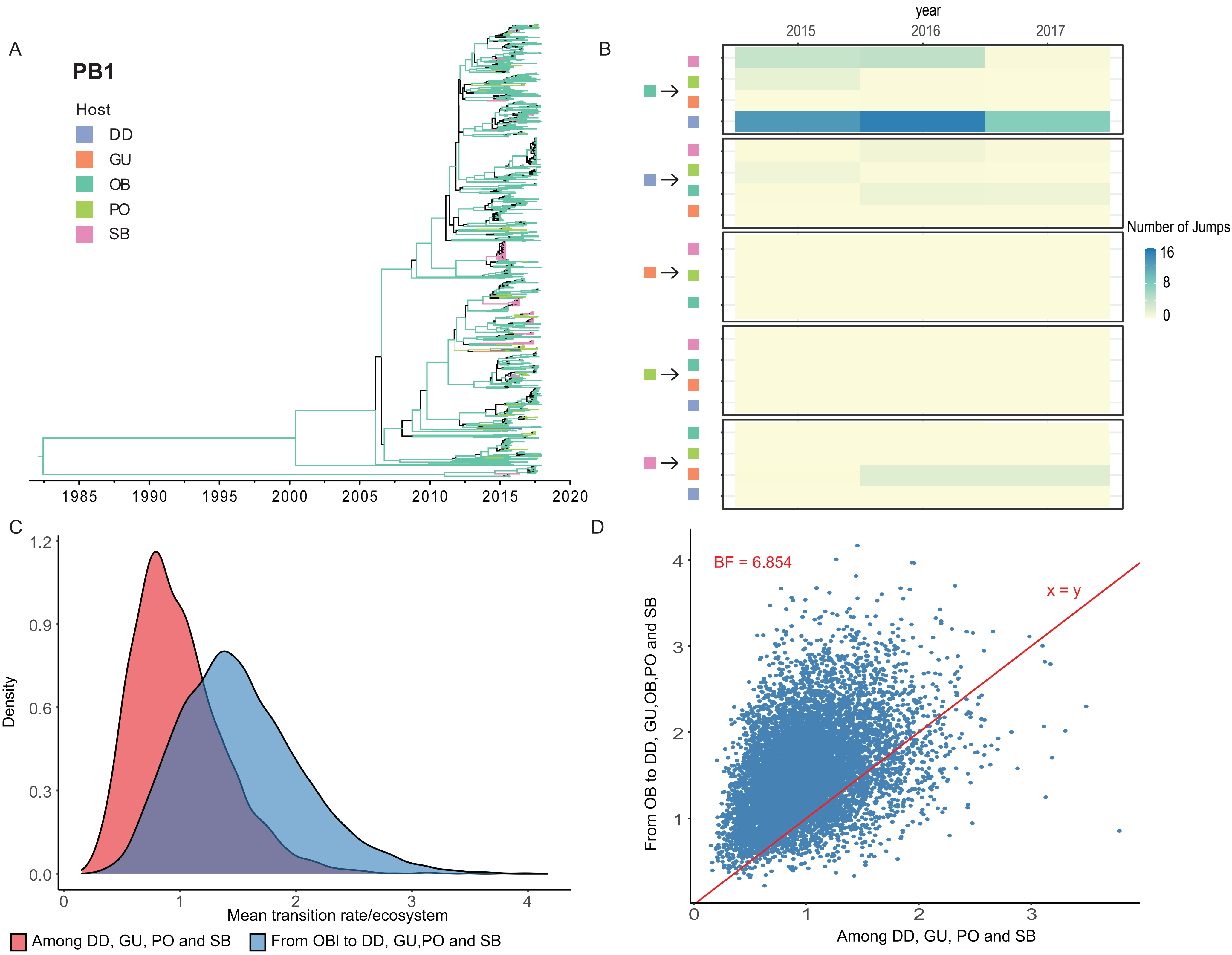

Bayesian phylogenetic tree (Panel A), heat map inferred number of Markov jumps among hosts groups (Panel B), plot of estimates for transitions from the other North American wild bird host group to northern Atlantic Flyway host groups as compared to strictly among northern Atlantic Flyway host groups (Panel C), and plot of ratios of posterior odds versus prior odds to infer differences in migration rate estimates between the other North American wild bird host group and northern Atlantic Flyway host groups as compared to strictly among northern Atlantic Flyway host groups (Panel D) for the PA gene segment.

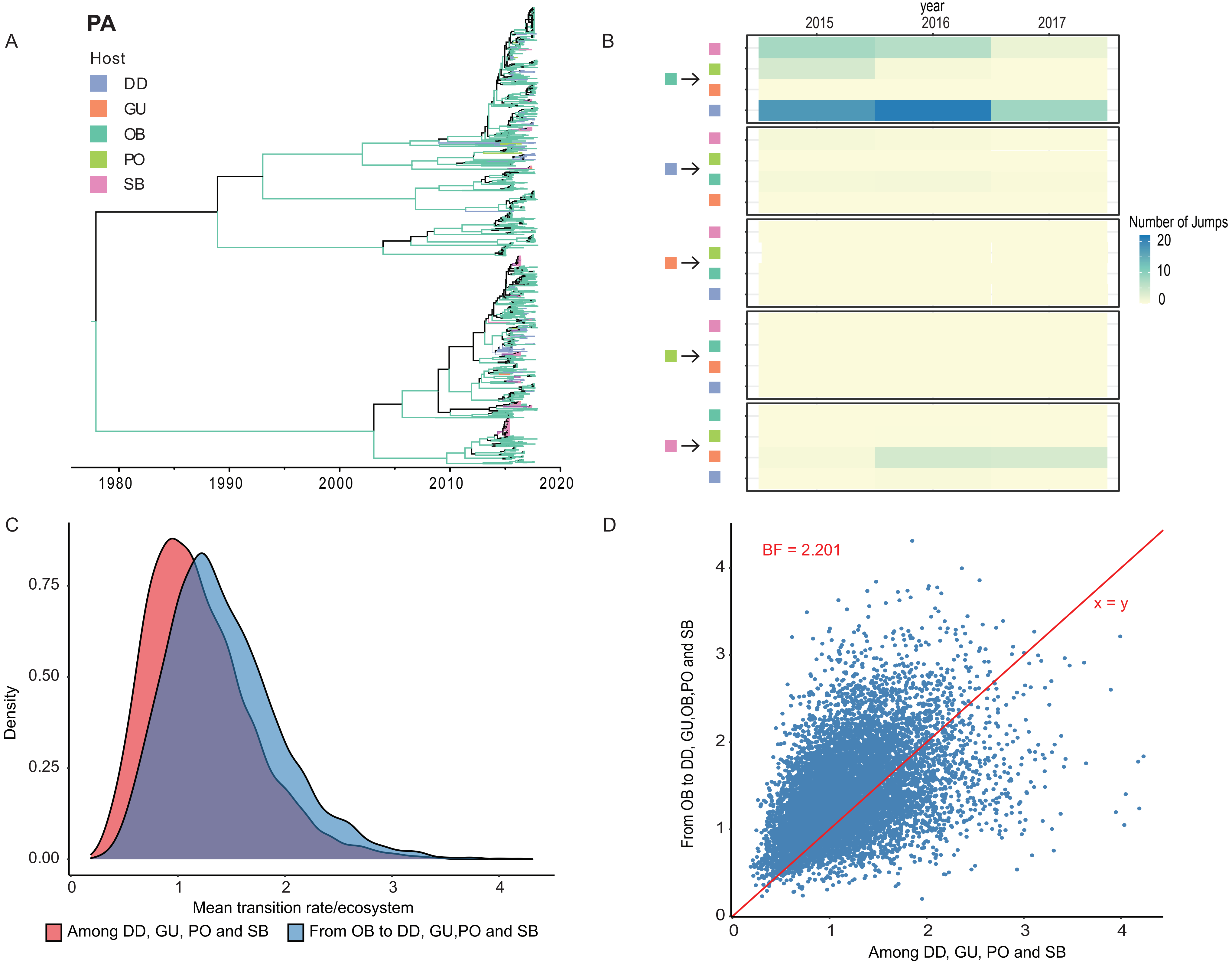

Bayesian phylogenetic tree (Panel A), heat map inferred number of Markov jumps among hosts groups (Panel B), plot of estimates for transitions from the other North American wild bird host group to northern Atlantic Flyway host groups as compared to strictly among northern Atlantic Flyway host groups (Panel C), and plot of ratios of posterior odds versus prior odds to infer differences in migration rate estimates between the other North American wild bird host group and northern Atlantic Flyway host groups as compared to strictly among northern Atlantic Flyway host groups (Panel D) for the NP gene segment.

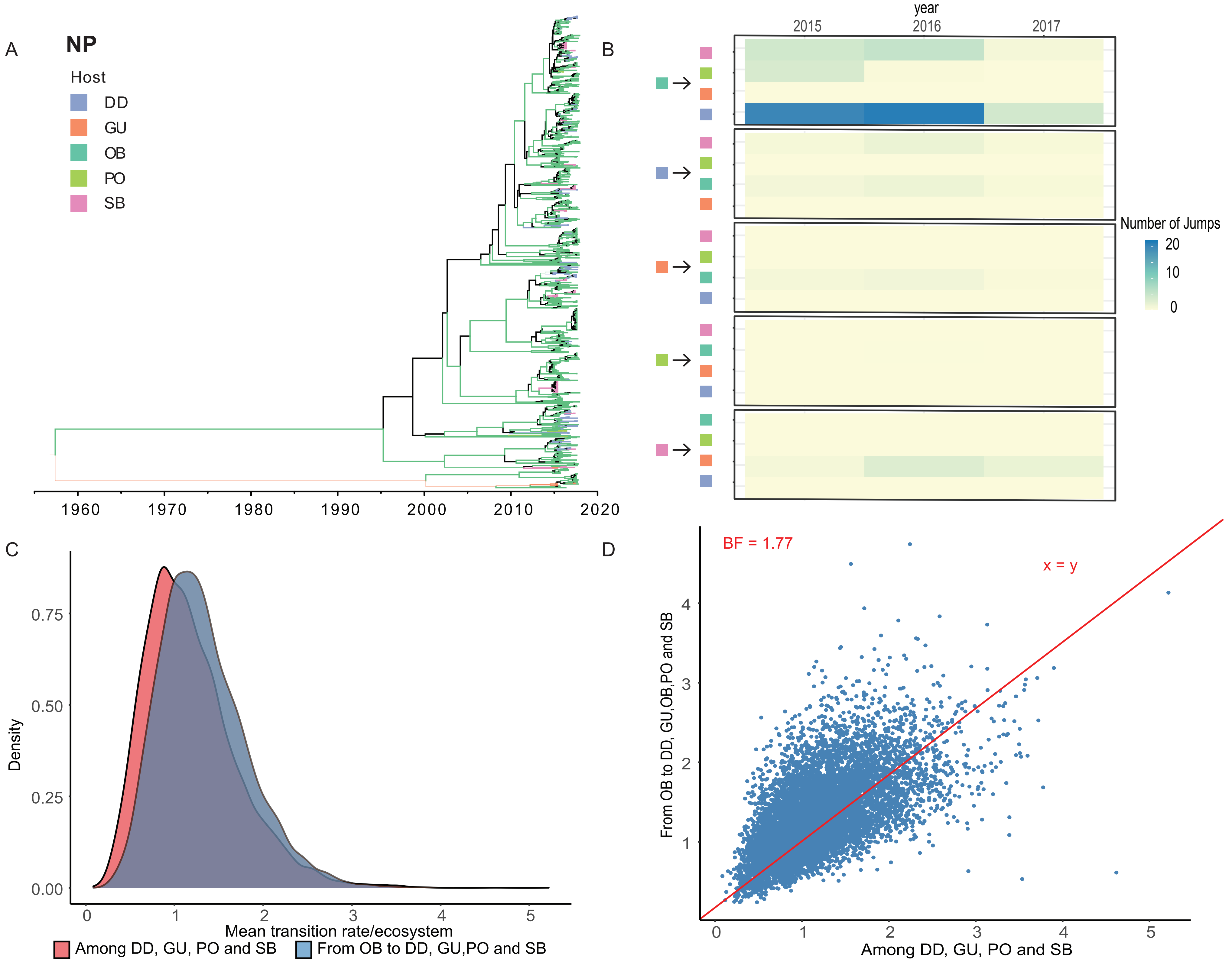

Bayesian phylogenetic tree (Panel A), heat map inferred number of Markov jumps among hosts groups (Panel B), plot of estimates for transitions from the other North American wild bird host group to northern Atlantic Flyway host groups as compared to strictly among northern Atlantic Flyway host groups (Panel C), and plot of ratios of posterior odds versus prior odds to infer differences in migration rate estimates between the other North American wild bird host group and northern Atlantic Flyway host groups as compared to strictly among northern Atlantic Flyway host groups (Panel D) for the M gene segment.

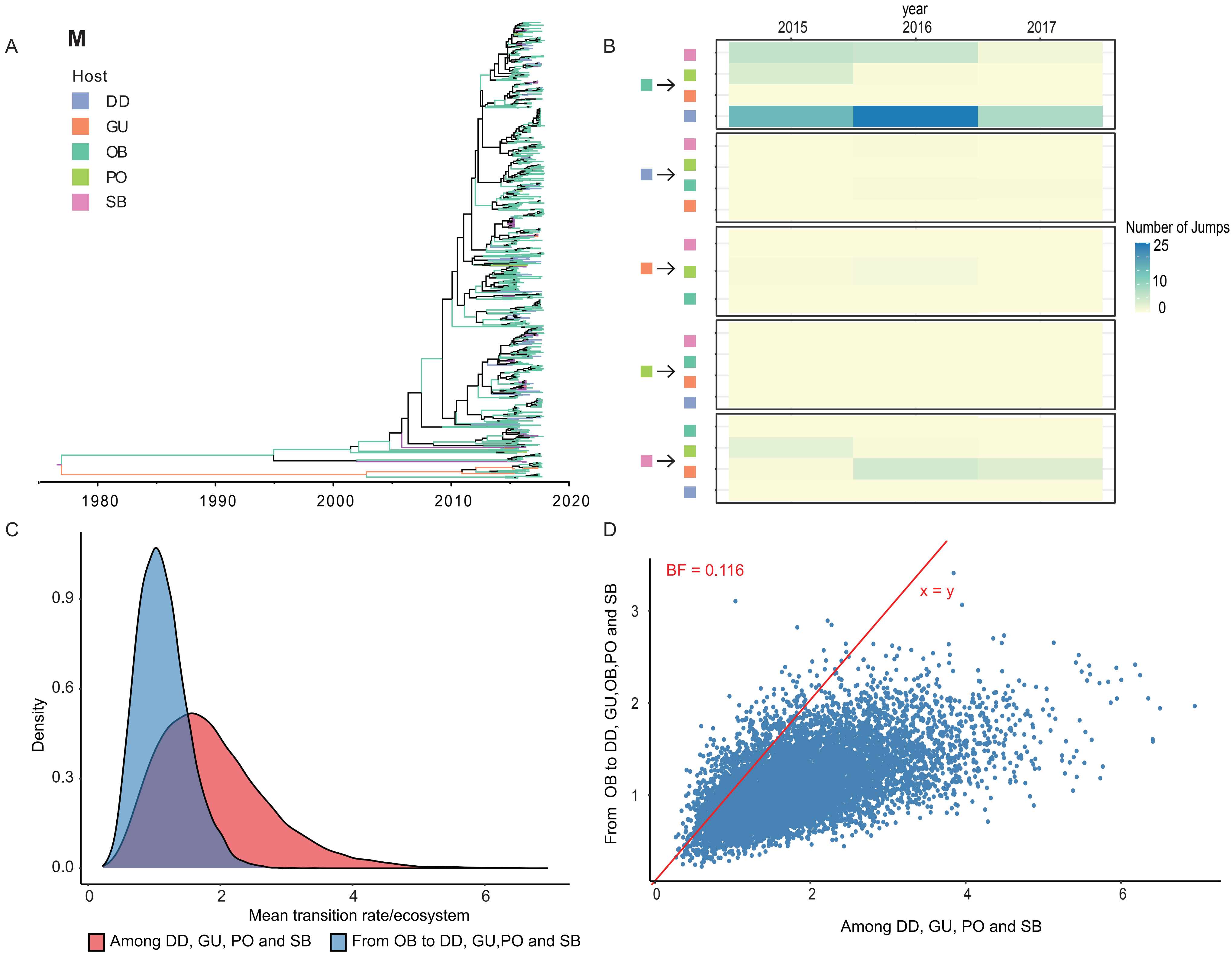

Bayesian phylogenetic tree (Panel A), heat map inferred number of Markov jumps among hosts groups (Panel B), plot of estimates for transitions from the other North American wild bird host group to northern Atlantic Flyway host groups as compared to strictly among northern Atlantic Flyway host groups (Panel C), and plot of ratios of posterior odds versus prior odds to infer differences in migration rate estimates between the other North American wild bird host group and northern Atlantic Flyway host groups as compared to strictly among northern Atlantic Flyway host groups (Panel D) for the NS gene segment.

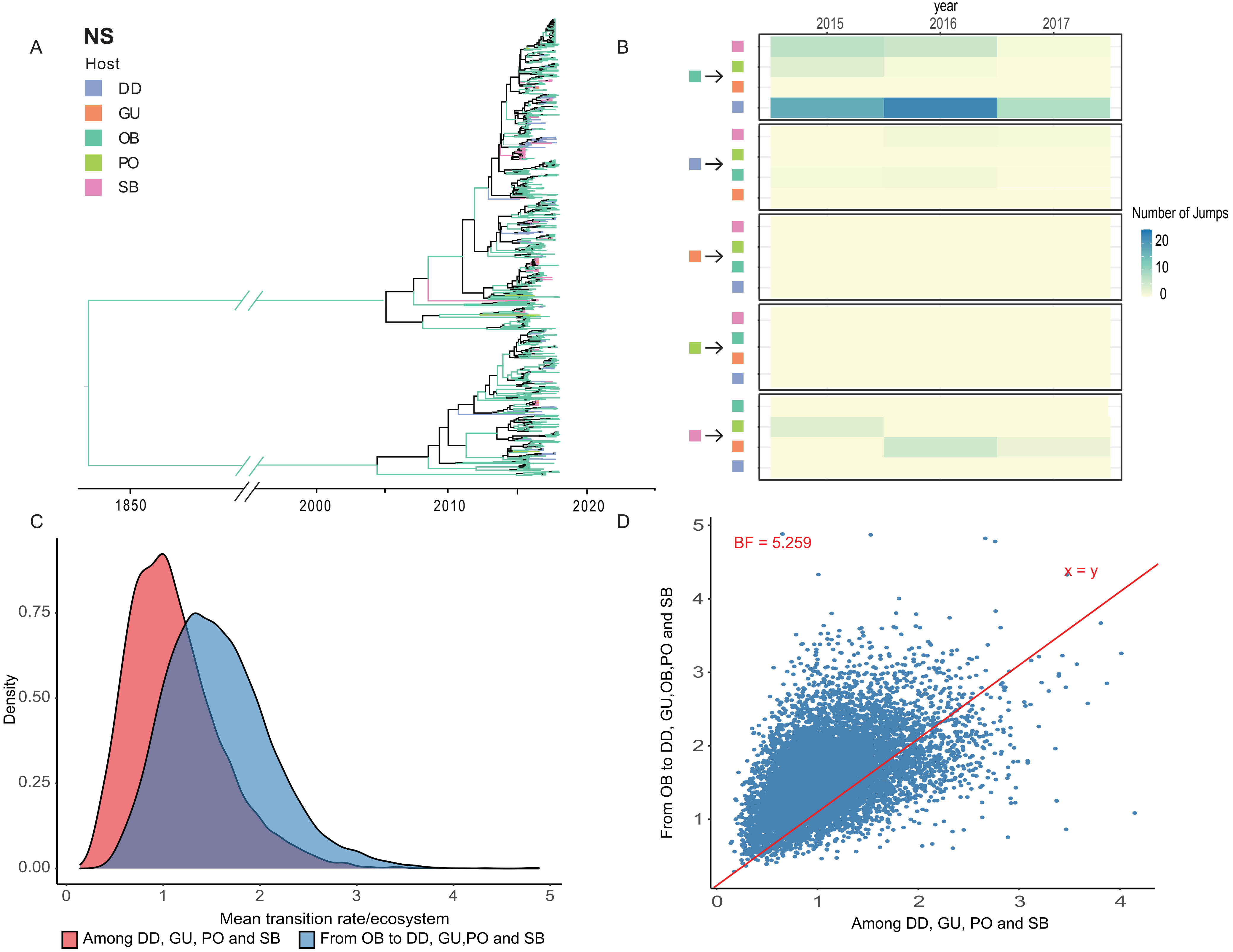

Bayesian phylogenetic tree (Panel A), heat map inferred number of Markov jumps among hosts groups (Panel B), plot of estimates for transitions from the other North American wild bird host group to northern Atlantic Flyway host groups as compared to strictly among northern Atlantic Flyway host groups (Panel C), and plot of ratios of posterior odds versus prior odds to infer differences in migration rate estimates between the other North American wild bird host group and northern Atlantic Flyway host groups as compared to strictly among northern Atlantic Flyway host groups (Panel D) for the H3 gene segment.

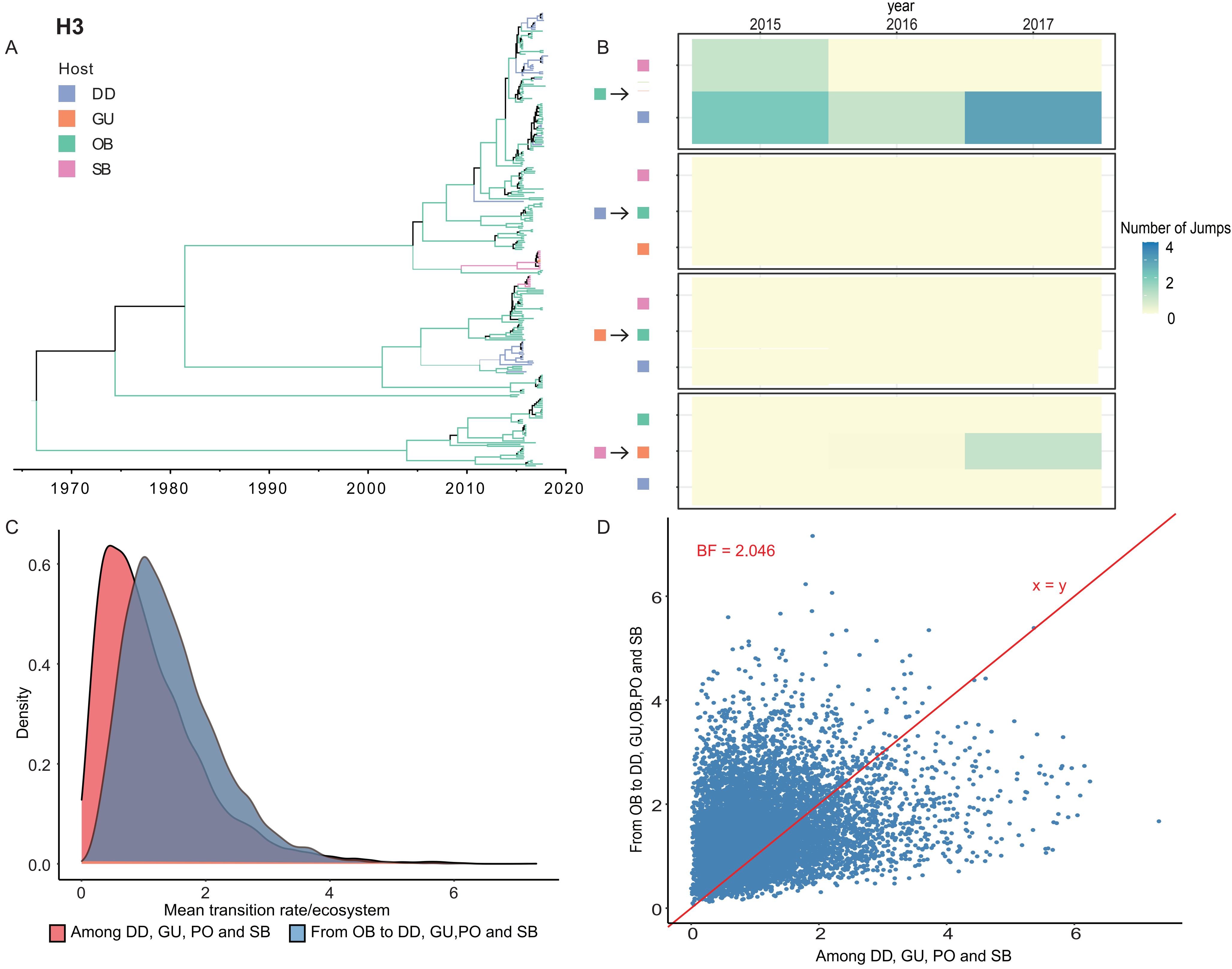

Bayesian phylogenetic tree (Panel A), heat map inferred number of Markov jumps among hosts groups (Panel B), plot of estimates for transitions from the other North American wild bird host group to northern Atlantic Flyway host groups as compared to strictly among northern Atlantic Flyway host groups (Panel C), and plot of ratios of posterior odds versus prior odds to infer differences in migration rate estimates between the other North American wild bird host group and northern Atlantic Flyway host groups as compared to strictly among northern Atlantic Flyway host groups (Panel D) for the H5 gene segment.

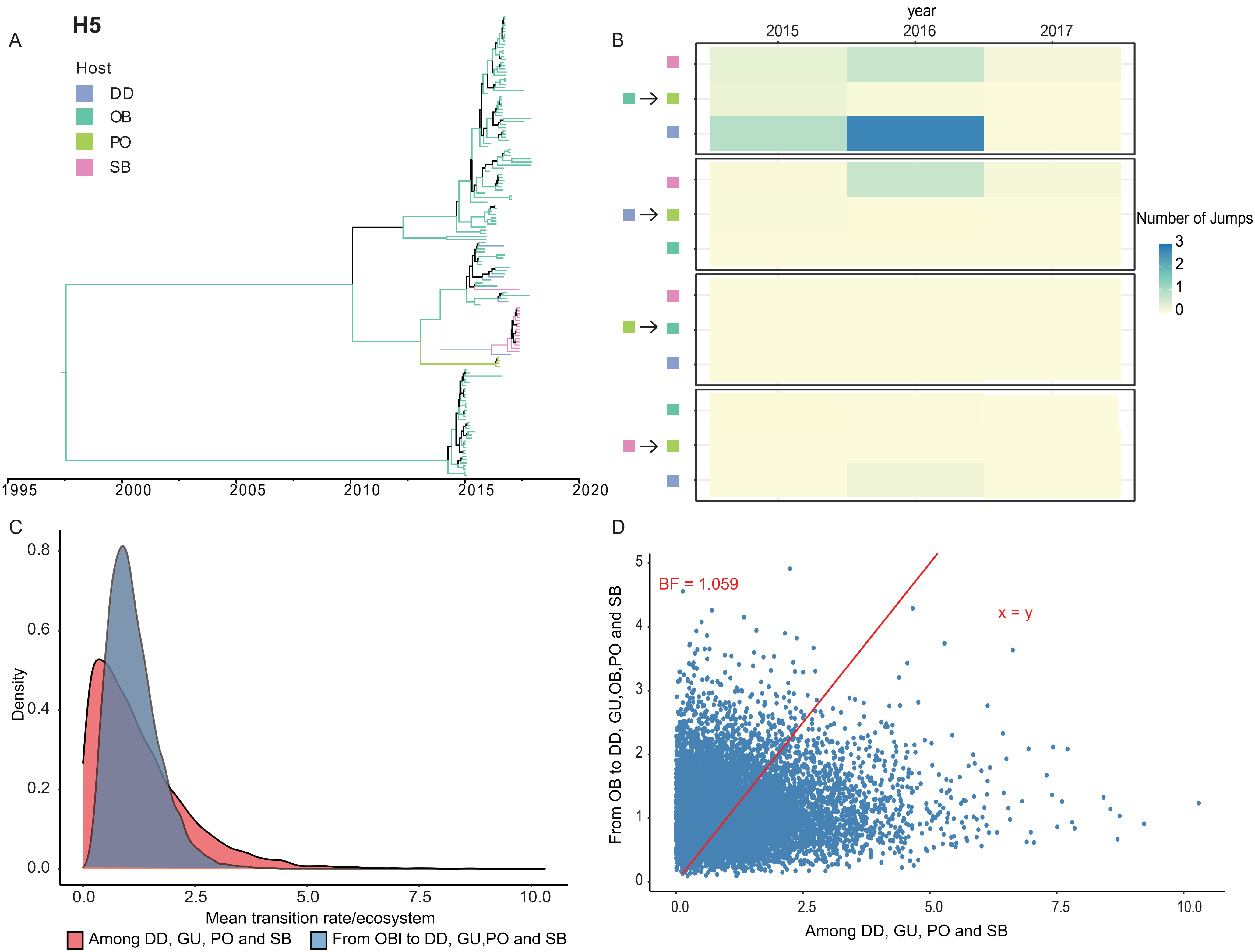

Bayesian phylogenetic tree (Panel A), heat map inferred number of Markov jumps among hosts groups (Panel B), plot of estimates for transitions from the other North American wild bird host group to northern Atlantic Flyway host groups as compared to strictly among northern Atlantic Flyway host groups (Panel C), and plot of ratios of posterior odds versus prior odds to infer differences in migration rate estimates between the other North American wild bird host group and northern Atlantic Flyway host groups as compared to strictly among northern Atlantic Flyway host groups (Panel D) for the N2 gene segment.

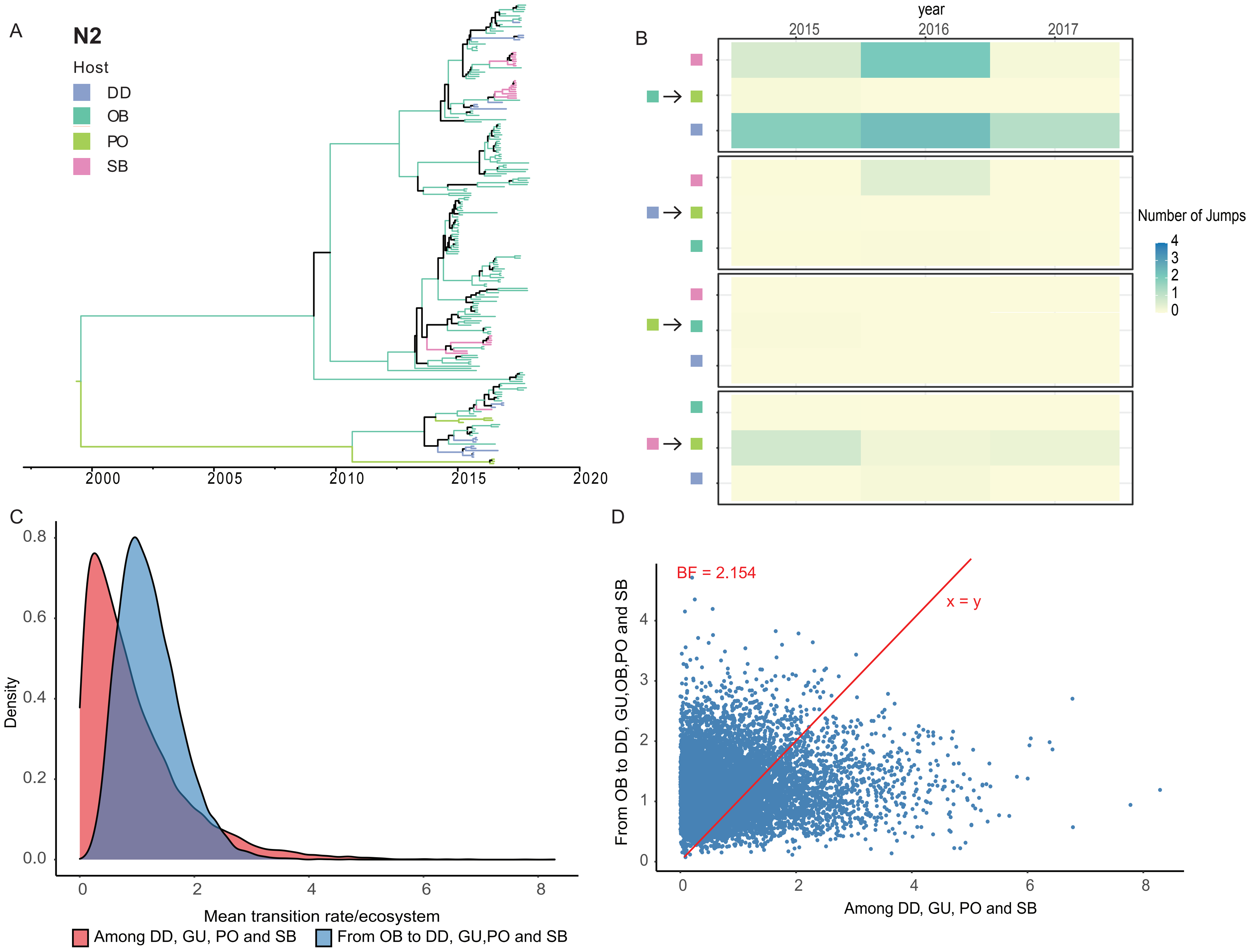

Bayesian phylogenetic tree (Panel A), heat map inferred number of Markov jumps among hosts groups (Panel B), plot of estimates for transitions from the other North American wild bird host group to northern Atlantic Flyway host groups as compared to strictly among northern Atlantic Flyway host groups (Panel C), and plot of ratios of posterior odds versus prior odds to infer differences in migration rate estimates between the other North American wild bird host group and northern Atlantic Flyway host groups as compared to strictly among northern Atlantic Flyway host groups (Panel D) for the N8 gene segment.

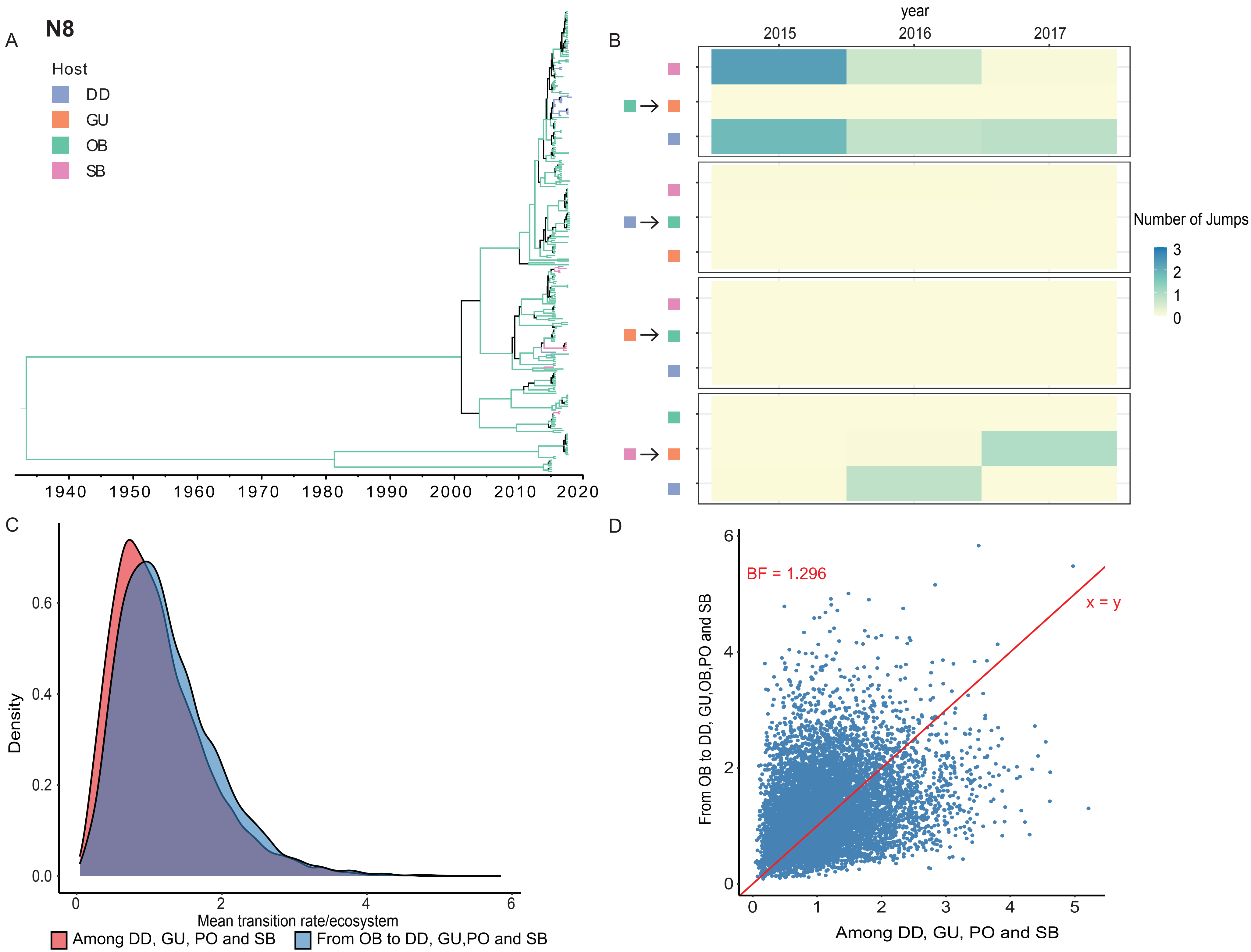

Bayesian phylogenetic tree (Panel A) and heat map inferred number of Markov jumps among hosts groups (Panel B) for the hemagglutinin H1 and neuraminidase N1 gene segments. Note that Panels C and D are not included as support for transmission within the Atlantic Flyway hosts was not determined (BF< 3) for these gene segments.

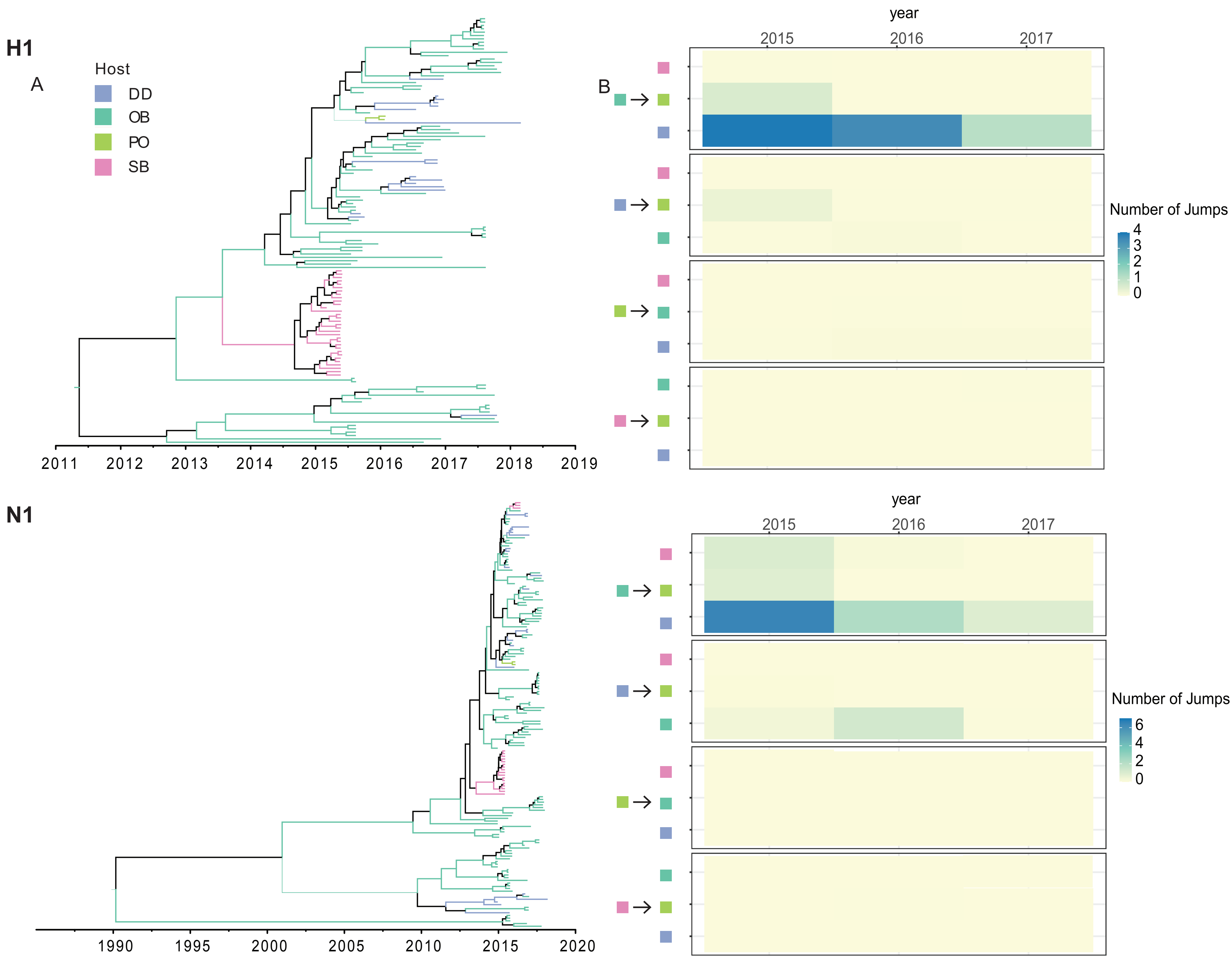

Supplement: S2 File — Bayesian phylogenetic tree (Panel A), heat map inferred number of Markov jumps among hosts groups (Panel B), plot of estimates for transitions from the other North American wild bird host group to northern Atlantic Flyway host groups as compared to strictly among northern Atlantic Flyway host groups (Panel C), and plot of ratios of posterior odds versus prior odds to infer differences in migration rate estimates between the other North American wild bird host group and northern Atlantic Flyway host groups as compared to strictly among northern Atlantic Flyway host groups (Panel D) for the PB1, PA, NP, M, and NS internal gene segments as well as hemagglutinin H3, and H5 and neuraminidase N2, and N8 gene segments. Note that for gene segments hemagglutinin H1 and neuraminidase N1, support for transmission within the Atlantic Flyway hosts was not determined (BF less than 3) and hence, panels C and D are not included. (PDF) [file ppat.1010605.s004.pdf]
