# Supplementary material for: Maintenance and dissemination of avian-origin influenza A virus within the northern Atlantic Flyway of North America
Source: PLoS Pathog. 2022 Jun 6;18(6):e1010605. doi: 10.1371/journal.ppat.1010605 (PMC9203021; doi:10.1371/journal.ppat.1010605)
Supplement: S3 File — This file lists the number of sequences per discrete host group and describes the sampling bias sensitivity analysis applied to explore whether the uneven sample sizes among host groups (particularly Other Birds; OB) greatly affected the phylogenetic analysis model results. Table A: List of segments for phylogenetic analysis per discrete host group. Table B: Tip-state randomization to examine ancestral root state probabilities. Fig A: Bar graph showing number of segments for phylogenetic analysis per discrete host group. (DOCX) [file ppat.1010605.s005.docx]

**Model sensitivity to sampling bias**

North American wild birds (OB) that are outside Atlantic Flyway were sampled much more intensively than Atlantic Flyway birds (Table S3, Figure S1). To investigate if our reconstruction was sensitive to data heterogeneity, we consider the prior expectation for the root discrete state (host) frequencies as a sensitivity analysis. If the discrete state distribution at the root is correlated with the host frequencies at the tips, we can expect that ancestral reconstruction throughout the entire phylogeny will be influenced by this tip-host sampling frequency. We randomized (“tip swap”) the host assignments at the tips throughout the MCMC procedure both with and without BSSVS analysis to investigate this possibility.

In an analysis of an unbiased sample, it was expected that the ancestral root state probabilities of a tip swap analysis should approximately equal each other (in the analysis of segment NS). In contrast, we observed a relatively high root state probabilities in the North American wild birds (OB) in the analyses of other segments, which suggests the sampling bias in the dataset. In the analysis for some gene segments (PB2, NP, M, N2), the ancestral root state posterior probabilities (pp) differ considerably between the tip swap analysis and the observed results (Table S4). For example, northern Atlantic Flyway gulls (GU) are estimated as the potential ancestral root state in the main analysis of PB2 gene segment (pp = 0.48), but the tip swap analysis estimates OB as the ancestral root state (pp (with BSSVS) = 0.687, pp (without BSSVS) = 0.746). In other words, based on the host sampling proportions alone, OB would be the most likely origin of IAV in all North American birds. Nevertheless, when the genetic data informs the analysis, the most likely origin of North America birds’ virus diversity was GU with a probability of 0.48. In the analysis for other gene segments, though the tip swap analysis and the main analysis predict the same host category as the most probable ancestral root state, the root state probabilities differ among the other categories. For instance, the most recent common ancestor of IAV within all North American birds was inferred to exist in North American wild birds in both the main (pp = 0.974) and tip swap (pp (with BSSVS) = 0.644, pp (without BSSVS) = 0.551) analyses using H1segment. The next most probable host for the most recent common ancestor of IAV within all North American birds was northern Atlantic Flyway dabbling ducks (DD) with a probability of 0.238 with BSSVS and 0.344 without BSSVS, according to the tip swap analysis, but only 0.002 probability in the main analysis. This suggests that while the ancestral state reconstruction is biased toward North American wild birds, the genetic data is driving the ancestral reconstruction of the categories of analytical interest. Overall, our sensitivity analysis indicates the sampling frequencies had little impact on the model inferences posteriori.

**Table A. List of segments for phylogenetic analysis per discrete host group.**

| Segment | DD | GU | OB | PO | SB |
| --- | --- | --- | --- | --- | --- |
| PB2 | 129 | 11 | 977 | 10 | 106 |
| PB1 | 91 | 5 | 927 | 9 | 109 |
| PA | 122 | 9 | 981 | 10 | 128 |
| NP | 110 | 12 | 937 | 10 | 90 |
| M | 96 | 13 | 793 | 8 | 70 |
| NS | 106 | 8 | 815 | 8 | 83 |
| H1 | 17 | 0 | 76 | 2 | 32 |
| H3 | 43 | 2 | 195 | 0 | 16 |
| H5 | 4 | 0 | 125 | 4 | 16 |
| N1 | 21 | 0 | 115 | 2 | 20 |
| N2 | 20 | 0 | 163 | 6 | 24 |
| N8 | 18 | 1 | 219 | 0 | 10 |

**Fig A. Bar graph showing number of segments for phylogenetic analysis per discrete host group.**

**Table B. Tip-state randomization to examine ancestral root state probabilities.**

| Segment | Host | Main analysis | Tip swap | Tip swap without BSSVS analysis |
| --- | --- | --- | --- | --- |
| PB2 | DD | 0.052 | 0.098 | 0.06 |
|  | GU | 0.48 | 0.105 | 0.138 |
|  | OB | 0.426 | 0.687 | 0.746 |
|  | PO | 0.003 | 0.033 | 0.024 |
|  | SB | 0.038 | 0.078 | 0.032 |
| PB1 | DD | 0.091 | 0.04 | 0.041 |
|  | GU | 0.013 | 0.034 | 0.04 |
|  | OB | 0.875 | 0.785 | 0.833 |
|  | PO | 0.015 | 0.051 | 0.041 |
|  | SB | 0.006 | 0.091 | 0.046 |
| PA | DD | 0.115 | 0.075 | 0.047 |
|  | GU | 0.01 | 0.023 | 0.021 |
|  | OB | 0.856 | 0.719 | 0.88 |
|  | PO | 0.009 | 0.036 | 0.019 |
|  | SB | 0.01 | 0.147 | 0.032 |
| NP | DD | 0.124 | 0.204 | 0.157 |
|  | GU | 0.446 | 0.076 | 0.101 |
|  | OB | 0.236 | 0.424 | 0.497 |
|  | PO | 0.015 | 0.109 | 0.085 |
|  | SB | 0.178 | 0.187 | 0.16 |
| M | DD | 0.026 | 0.241 | 0.196 |
|  | GU | 0.376 | 0.033 | 0.063 |
|  | OB | 0.18 | 0.464 | 0.552 |
|  | PO | 0.01 | 0.056 | 0.061 |
|  | SB | 0.407 | 0.206 | 0.128 |
| NS | DD | 0.307 | 0.278 | 0.217 |
|  | GU | 0.105 | 0.09 | 0.182 |
|  | OB | 0.269 | 0.209 | 0.21 |
|  | PO | 0.154 | 0.147 | 0.185 |
|  | SB | 0.165 | 0.277 | 0.206 |
| H1 | DD | 0.002 | 0.238 | 0.344 |
|  | OB | 0.974 | 0.644 | 0.551 |
|  | PO | 0.002 | 0.044 | 0.03 |
|  | SB | 0.022 | 0.074 | 0.076 |
| H3 | DD | 0.009 | 0.118 | 0.085 |
|  | GU | 0.013 | 0.063 | 0.028 |
|  | OB | 0.973 | 0.754 | 0.848 |
|  | SB | 0.005 | 0.065 | 0.039 |
| H5 | DD | 0.026 | 0.045 | 0.052 |
|  | OB | 0.865 | 0.784 | 0.824 |
|  | PO | 0.075 | 0.072 | 0.054 |
|  | SB | 0.034 | 0.099 | 0.07 |
| N1 | DD | 0.25 | 0.198 | 0.364 |
|  | OB | 0.687 | 0.554 | 0.394 |
|  | PO | 0.032 | 0.034 | 0.038 |
|  | SB | 0.031 | 0.214 | 0.204 |
| N2 | DD | 0.026 | 0.052 | 0.108 |
|  | OB | 0.43 | 0.66 | 0.704 |
|  | PO | 0.485 | 0.068 | 0.059 |
|  | SB | 0.059 | 0.221 | 0.13 |
| N8 | DD | 0.051 | 0.108 | 0.09 |
|  | GU | 0.041 | 0.087 | 0.074 |
|  | OB | 0.878 | 0.689 | 0.731 |
|  | SB | 0.029 | 0.116 | 0.104 |
