# Supplementary material for: Maintenance and dissemination of avian-origin influenza A virus within the northern Atlantic Flyway of North America
Source: PLoS Pathog. 2022 Jun 6;18(6):e1010605. doi: 10.1371/journal.ppat.1010605 (PMC9203021; doi:10.1371/journal.ppat.1010605)
Supplement: S4 File — This file contains phylogenetic trees printed and attached in a PDF format for those who do not have FigTree or other software to view the treeplots. (PDF) [file ppat.1010605.s006.pdf]

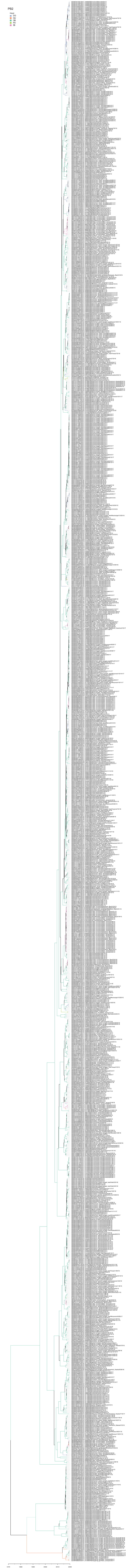
PB2
Host
DD
GU
OB
PO
SB
1970
1980
1990
2000
2010
2020

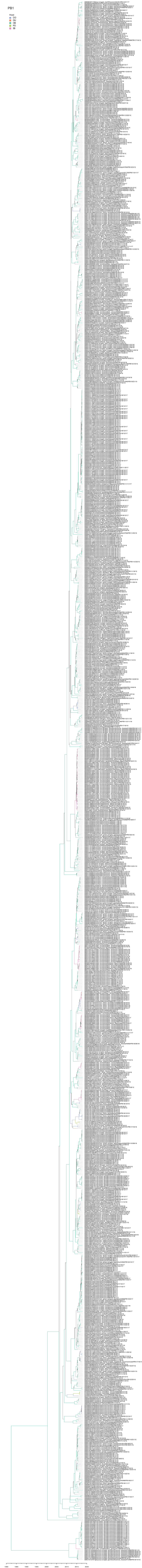
PB1
Host
DD
GU
OB
PO
SB
1980
1985
1990
1995
2000
2005
2010
2015
2020

PA

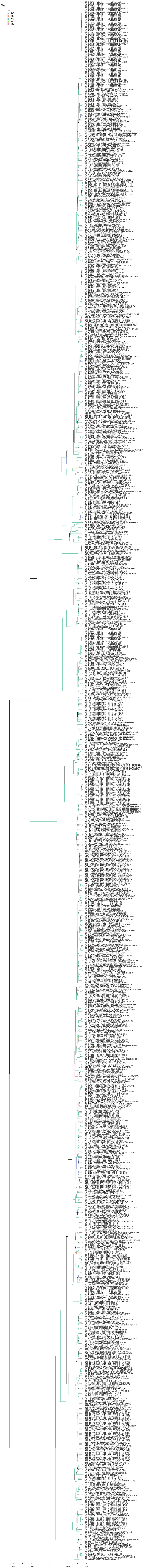

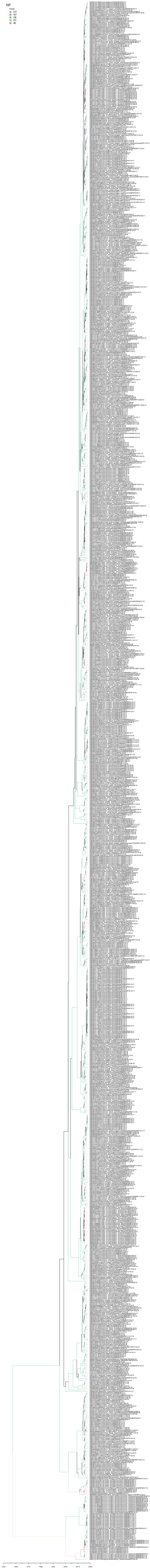
NP
Host
DD
GU
OB
PO
SB
1950
1960
1970
1980
1990
2000
2010
2020

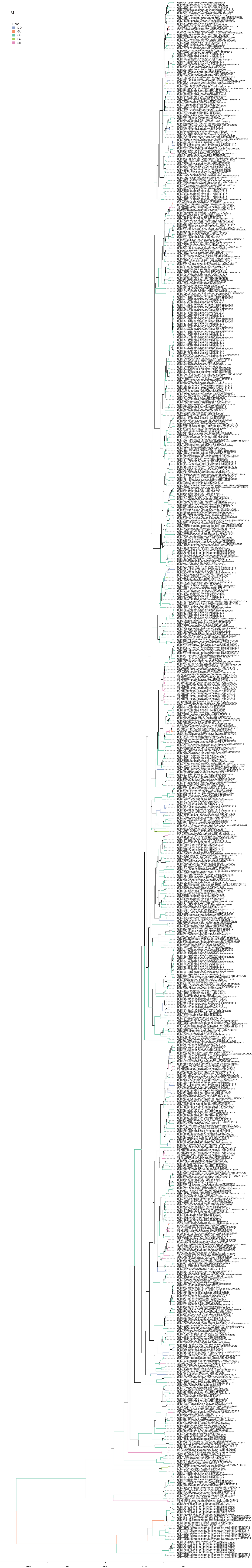
M
Host
DD
GU
OB
PO
SB
1980
1990
2000
2010
2020

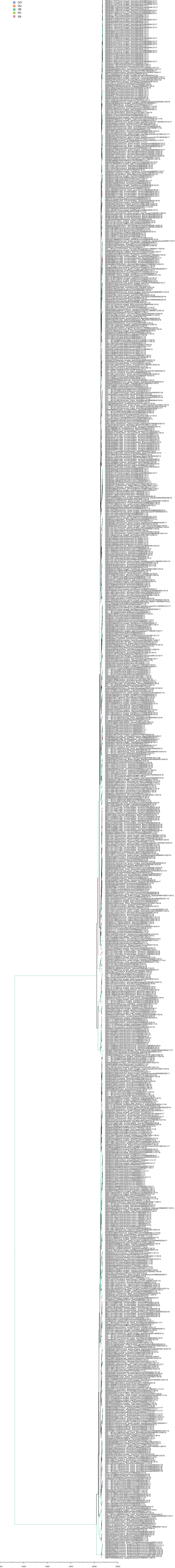
NS
Host
DD
GU
OB
PO
SB
1850
1900
1950
2000
2050

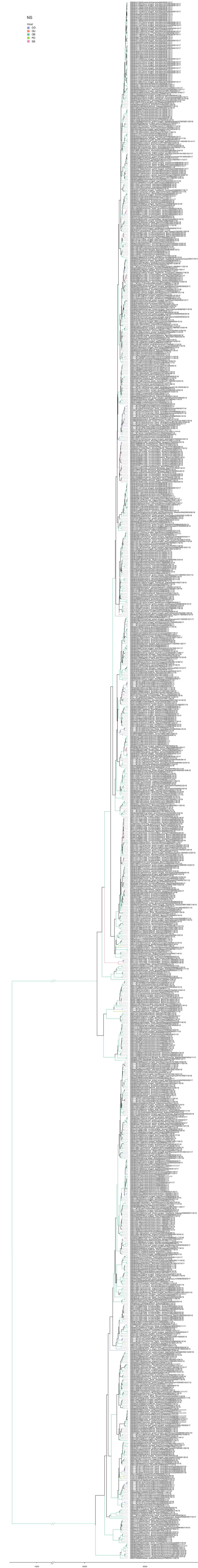
NS
Host
DD
GU
OB
PO
SB
1850
2000
2025

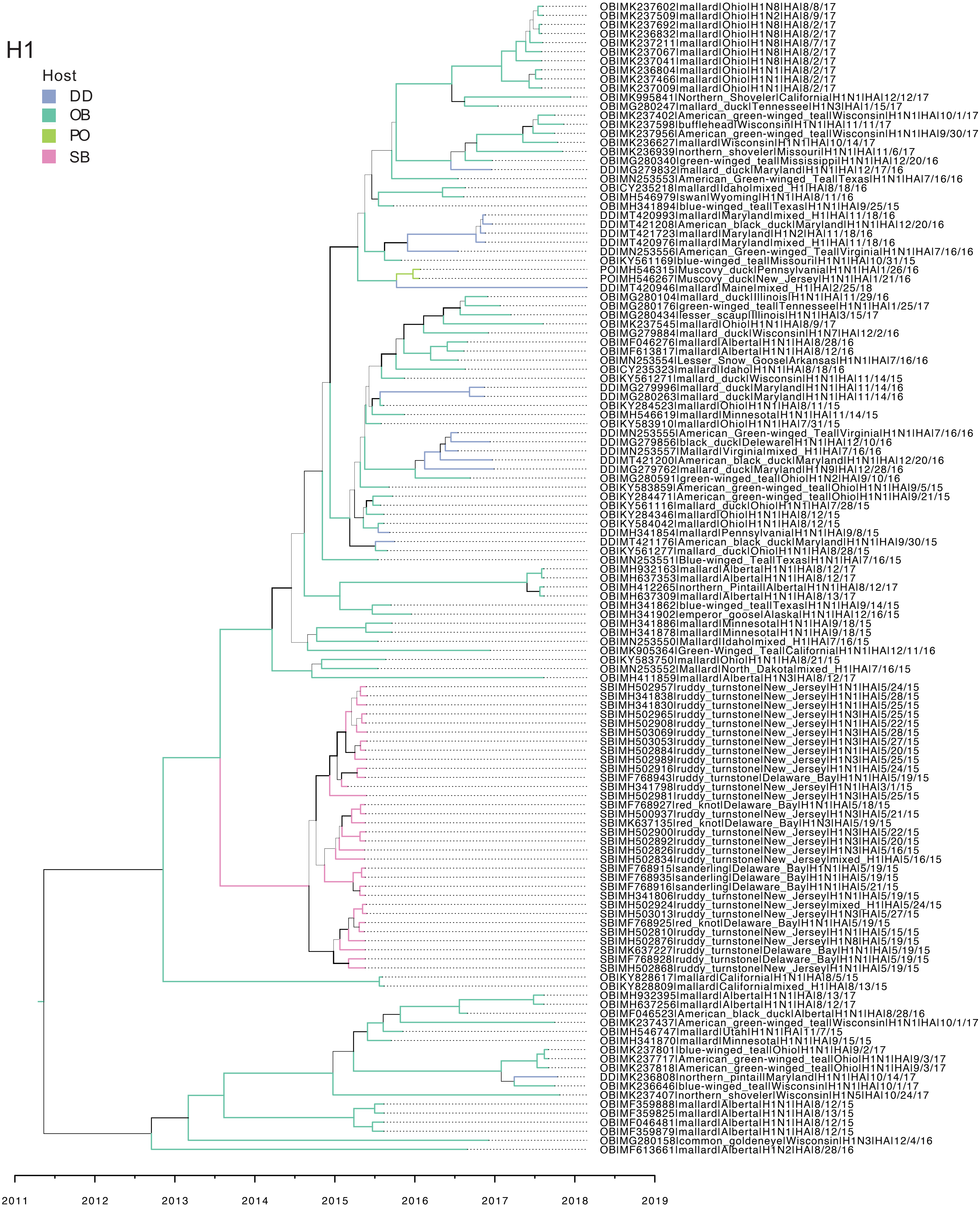
H1
Host
DD
OB
PO
SB
OB|MK237602|mallard|Ohio|H1N8|HA|8/8/17
OB|MK237509|mallard|Ohio|H1N2|HA|8/9/17
OB|MK237692|mallard|Ohio|H1N8|HA|8/2/17
OB|MK236832|mallard|Ohio|H1N8|HA|8/2/17
OB|MK237211|mallard|Ohio|H1N8|HA|8/7/17
OB|MK237067|mallard|Ohio|H1N8|HA|8/2/17
OB|MK237041|mallard|Ohio|H1N8|HA|8/2/17
OB|MK236804|mallard|Ohio|H1N1|HA|8/2/17
OB|MK237466|mallard|Ohio|H1N1|HA|8/2/17
OB|MK237009|mallard|Ohio|H1N1|HA|8/2/17
OB|MK995841|Northern_Shoveler|California|H1N8|HA|12/12/17
OB|MG280247|mallard_duck|Tennessee|H1N3|HA|1/15/17
OB|MK237402|American_green-winged_teal|Wisconsin|H1N1|HA|10/1/17
OB|MK237598|bufflehead|Wisconsin|H1N1|HA|11/11/17
OB|MK237956|American_green-winged_teal|Wisconsin|H1N1|HA|9/30/17
OB|MK236627|mallard|Wisconsin|H1N1|HA|10/14/17
OB|MK236939|northern_shoveler|Missouri|H1N1|HA|11/6/17
OB|MG280340|green-winged_teal|Mississippi|H1N1|HA|12/20/16
DD|MG279832|mallard_duck|Maryland|H1N1|HA|12/17/16
OB|MN253553|American_Green-winged_Teal|Texas|H1N1|HA|7/16/16
OB|CY235218|mallard|Idaho|mixed_H1|HA|8/18/16
OB|MH546979|swan|Wyoming|H1N1|HA|8/11/16
OB|MH341894|blue-winged_teal|Texas|H1N1|HA|9/25/15
DD|MT420993|mallard|Maryland|mixed_H1|HA|11/18/16
DD|MT421208|American_black_duck|Maryland|H1N1|HA|12/20/16
DD|MT421723|mallard|Maryland|H1N2|HA|11/18/16
DD|MT420976|mallard|Maryland|mixed_H1|HA|11/18/16
DD|MN253556|American_Green-winged_Teal|Virginia|H1N1|HA|7/16/16
OB|KY561169|blue-winged_teal|Missouri|H1N1|HA|10/31/15
PO|MH546315|Muscovy_duck|Pennsylvania|H1N1|HA|1/26/16
PO|MH546267|Muscovy_duck|New_Jersey|H1N1|HA|1/21/16
DD|MT420946|mallard|Maine|mixed_H1|HA|2/25/18
OB|MG280104|mallard_duck|Illinois|H1N1|HA|11/29/16
OB|MG280176|green-winged_teal|Tennessee|H1N1|HA|1/25/17
OB|MG280434|lesser_scaup|Illinois|H1N1|HA|3/15/17
OB|MK237545|mallard|Ohio|H1N1|HA|8/9/17
OB|MG279884|mallard_duck|Wisconsin|H1N7|HA|12/2/16
OB|MF046276|mallard|Alberta|H1N1|HA|8/28/16
OB|MF613817|mallard|Alberta|H1N1|HA|8/12/16
OB|MN253554|Lesser_Snow_Goose|Arkansas|H1N1|HA|7/16/16
OB|CY235323|mallard|Idaho|H1N1|HA|8/18/16
OB|KY561271|mallard_duck|Wisconsin|H1N1|HA|11/14/15
DD|MG279996|mallard_duck|Maryland|H1N1|HA|11/14/16
DD|MG280263|mallard_duck|Maryland|H1N1|HA|11/14/16
OB|KY284523|mallard|Ohio|H1N1|HA|8/11/15
OB|MH546619|mallard|Minnesota|H1N1|HA|11/14/15
OB|KY583910|mallard|Ohio|H1N1|HA|7/31/15
DD|MN253555|American_Green-winged_Teal|Virginia|H1N1|HA|7/16/16
DD|MG279856|black_duck|Deleware|H1N1|HA|12/10/16
DD|MN253557|Mallard|Virginia|mixed_H1|HA|7/16/16
DD|MT421200|American_black_duck|Maryland|H1N1|HA|12/20/16
DD|MG279762|mallard_duck|Maryland|H1N9|HA|12/28/16
OB|MG280591|green-winged_teal|Ohio|H1N2|HA|9/10/16
OB|KY583859|American_green-winged_teal|Ohio|H1N1|HA|9/5/15
OB|KY284471|American_green-winged_teal|Ohio|H1N1|HA|9/21/15
OB|KY561116|mallard_duck|Ohio|H1N1|HA|7/28/15
OB|KY284346|mallard|Ohio|H1N1|HA|8/12/15
OB|KY584042|mallard|Ohio|H1N1|HA|8/12/15
DD|MH341854|mallard|Pennsylvania|H1N1|HA|9/8/15
DD|MT421176|American_black_duck|Maryland|H1N1|HA|9/30/15
OB|KY561277|mallard_duck|Ohio|H1N1|HA|8/28/15
OB|MN253551|Blue-winged_Teal|Texas|H1N1|HA|7/16/15
OB|MH932163|mallard|Alberta|H1N1|HA|8/12/17
OB|MH637353|mallard|Alberta|H1N1|HA|8/12/17
OB|MH412265|northern_Pintail|Alberta|H1N1|HA|8/12/17
OB|MH637309|mallard|Alberta|H1N1|HA|8/13/17
OB|MH341862|blue-winged_teal|Texas|H1N1|HA|9/14/15
OB|MH341902|emperor_goose|Alaska|H1N1|HA|12/16/15
OB|MH341886|mallard|Minnesota|H1N1|HA|9/18/15
OB|MH341878|mallard|Minnesota|H1N1|HA|9/18/15
OB|MN253550|Mallard|Idaho|mixed_H1|HA|7/16/15
OB|MK905364|Green-Winged_Teal|California|H1N1|HA|12/11/16
OB|KY583750|mallard|Ohio|H1N1|HA|8/21/15
OB|MN253552|Mallard|North_Dakota|mixed_H1|HA|7/16/15
OB|MH411859|mallard|Alberta|H1N3|HA|8/12/17
SB|MH502957|ruddy_turnstone|New_Jersey|H1N1|HA|5/24/15
SB|MH341838|ruddy_turnstone|New_Jersey|H1N1|HA|5/28/15
SB|MH341830|ruddy_turnstone|New_Jersey|H1N1|HA|5/25/15
SB|MH502965|ruddy_turnstone|New_Jersey|H1N3|HA|5/25/15
SB|MH502908|ruddy_turnstone|New_Jersey|H1N1|HA|5/22/15
SB|MH503069|ruddy_turnstone|New_Jersey|H1N3|HA|5/28/15
SB|MH503053|ruddy_turnstone|New_Jersey|H1N3|HA|5/27/15
SB|MH502884|ruddy_turnstone|New_Jersey|H1N1|HA|5/20/15
SB|MH502989|ruddy_turnstone|New_Jersey|H1N3|HA|5/25/15
SB|MH502916|ruddy_turnstone|New_Jersey|H1N1|HA|5/24/15
SB|MF768943|ruddy_turnstone|Delaware_Bay|H1N1|HA|5/19/15
SB|MH341798|ruddy_turnstone|New_Jersey|H1N1|HA|3/1/15
SB|MH502981|ruddy_turnstone|New_Jersey|H1N3|HA|5/25/15
SB|MF768927|red_knot|Delaware_Bay|H1N1|HA|5/18/15
SB|MH500937|ruddy_turnstone|New_Jersey|H1N3|HA|5/21/15
SB|MK637135|red_knot|Delaware_Bay|H1N3|HA|5/19/15
SB|MH502900|ruddy_turnstone|New_Jersey|H1N3|HA|5/22/15
SB|MH502892|ruddy_turnstone|New_Jersey|H1N3|HA|5/20/15
SB|MH502826|ruddy_turnstone|New_Jersey|H1N3|HA|5/16/15
SB|MH502834|ruddy_turnstone|New_Jersey|mixed_H1|HA|5/16/15
SB|MF768915|sanderling|Delaware_Bay|H1N1|HA|5/19/15
SB|MF768935|sanderling|Delaware_Bay|H1N1|HA|5/19/15
SB|MF768916|sanderling|Delaware_Bay|H1N1|HA|5/21/15
SB|MH341806|ruddy_turnstone|New_Jersey|H1N1|HA|5/19/15
SB|MH502924|ruddy_turnstone|New_Jersey|mixed_H1|HA|5/24/15
SB|MH503013|ruddy_turnstone|New_Jersey|H1N3|HA|5/27/15
SB|MF768925|red_knot|Delaware_Bay|H1N1|HA|5/19/15
SB|MH502810|ruddy_turnstone|New_Jersey|H1N1|HA|5/15/15
SB|MH502876|ruddy_turnstone|New_Jersey|H1N8|HA|5/19/15
SB|MK637227|ruddy_turnstone|Delaware_Bay|H1N1|HA|5/19/15
SB|MF768928|ruddy_turnstone|Delaware_Bay|H1N1|HA|5/19/15
SB|MH502868|ruddy_turnstone|New_Jersey|H1N1|HA|5/19/15
OB|KY828617|mallard|California|H1N1|HA|8/5/15
OB|KY828809|mallard|California|mixed_H1|HA|8/13/15
OB|MH932395|mallard|Alberta|H1N1|HA|8/13/17
OB|MH637256|mallard|Alberta|H1N1|HA|8/12/17
OB|MF046523|American_black_duck|Alberta|H1N1|HA|8/28/16
OB|MK237437|American_green-winged_teal|Wisconsin|H1N1|HA|10/1/17
OB|MH546747|mallard|Utah|H1N1|HA|11/7/15
OB|MH341870|mallard|Minnesota|H1N1|HA|9/15/15
OB|MK237801|blue-winged_teal|Ohio|H1N1|HA|9/2/17
OB|MK237717|American_green-winged_teal|Ohio|H1N1|HA|9/3/17
OB|MK237818|American_green-winged_teal|Ohio|H1N1|HA|9/3/17
DD|MK236808|northern_pintail|Maryland|H1N1|HA|10/14/17
OB|MK236646|blue-winged_teal|Wisconsin|H1N1|HA|10/1/17
OB|MK237407|northern_shoveler|Wisconsin|H1N5|HA|10/24/17
OB|MF359888|mallard|Alberta|H1N1|HA|8/12/15
OB|MF359825|mallard|Alberta|H1N1|HA|8/13/15
OB|MF046481|mallard|Alberta|H1N1|HA|8/12/15
OB|MF359879|mallard|Alberta|H1N1|HA|8/12/15
OB|MG280158|common_goldeneye|Wisconsin|H1N3|HA|12/4/16
OB|MF613661|mallard|Alberta|H1N2|HA|8/28/16
2011
2012
2013
2014
2015
2016
2017
2018
2019

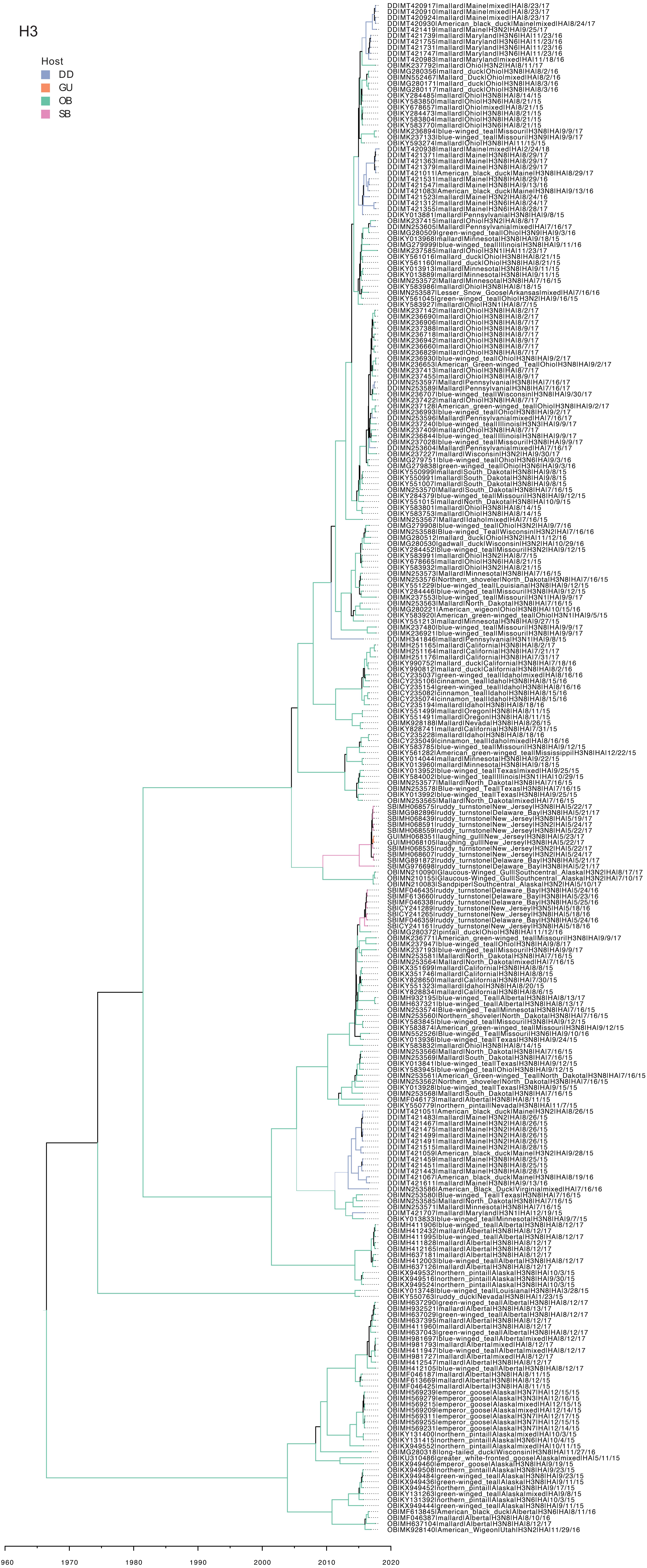

H3
Host
DD
GU
OB
SB
1960
1970
1980
1990
2000
2010
2020

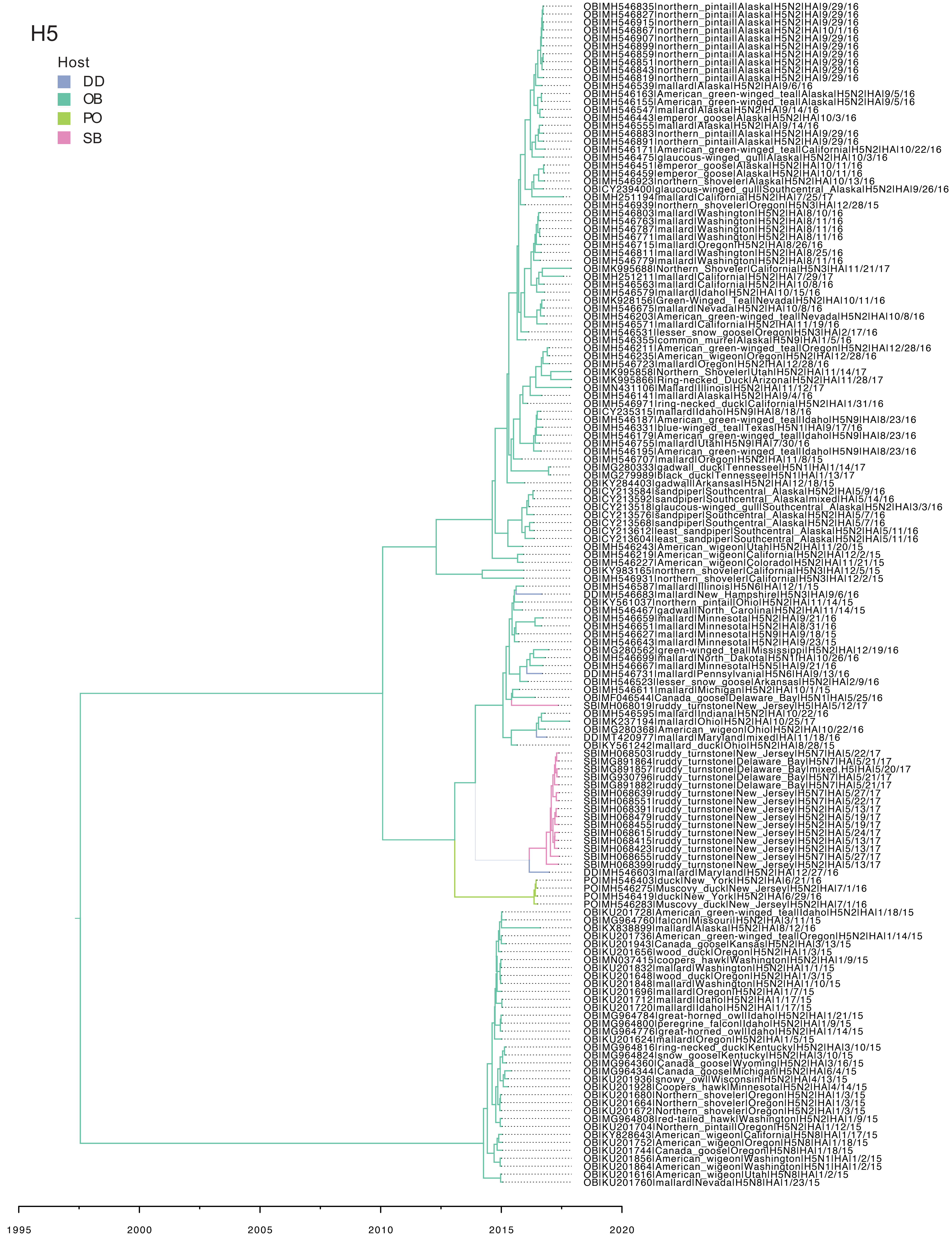

H5
Host
DD
OB
PO
SB
OB|MH546835|northern_pintail|Alaska|H5N2|HA|9/29/16
OB|MH546827|northern_pintail|Alaska|H5N2|HA|9/29/16
OB|MH546915|northern_pintail|Alaska|H5N2|HA|9/29/16
OB|MH546867|northern_pintail|Alaska|H5N2|HA|10/1/16
OB|MH546907|northern_pintail|Alaska|H5N2|HA|9/29/16
OB|MH546899|northern_pintail|Alaska|H5N2|HA|9/29/16
OB|MH546859|northern_pintail|Alaska|H5N2|HA|9/29/16
OB|MH546851|northern_pintail|Alaska|H5N2|HA|9/29/16
OB|MH546843|northern_pintail|Alaska|H5N2|HA|9/29/16
OB|MH546819|northern_pintail|Alaska|H5N2|HA|9/29/16
OB|MH546539|mallard|Alaska|H5N2|HA|9/6/16
OB|MH546163|American_green-winged_teal|Alaska|H5N2|HA|9/5/16
OB|MH546155|American_green-winged_teal|Alaska|H5N2|HA|9/5/16
OB|MH546547|mallard|Alaska|H5N2|HA|9/14/16
OB|MH546443|emperor_goose|Alaska|H5N2|HA|10/3/16
OB|MH546555|mallard|Alaska|H5N2|HA|9/14/16
OB|MH546883|northern_pintail|Alaska|H5N2|HA|9/29/16
OB|MH546891|northern_pintail|Alaska|H5N2|HA|9/29/16
OB|MH546171|American_green-winged_teal|California|H5N2|HA|10/22/16
OB|MH546475|glaucous-winged_gull|Alaska|H5N2|HA|10/3/16
OB|MH546451|emperor_goose|Alaska|H5N2|HA|10/11/16
OB|MH546459|emperor_goose|Alaska|H5N2|HA|10/11/16
OB|MH546923|northern_shoveler|Alaska|H5N2|HA|10/13/16
OB|CY239400|glaucous-winged_gull|Southcentral_Alaska|H5N2|HA|9/26/16
OB|MH251194|mallard|California|H5N2|HA|7/25/17
OB|MH546939|northern_shoveler|Oregon|H5N3|HA|12/28/15
OB|MH546803|mallard|Washington|H5N2|HA|8/10/16
OB|MH546763|mallard|Washington|H5N2|HA|8/11/16
OB|MH546787|mallard|Washington|H5N2|HA|8/11/16
OB|MH546771|mallard|Washington|H5N2|HA|8/11/16
OB|MH546715|mallard|Oregon|H5N2|HA|8/26/16
OB|MH546811|mallard|Washington|H5N2|HA|8/25/16
OB|MH546779|mallard|Washington|H5N2|HA|8/11/16
OB|MK995688|Northern_Shoveler|California|H5N3|HA|11/21/17
OB|MH251211|mallard|California|H5N2|HA|7/29/17
OB|MH546563|mallard|California|H5N2|HA|10/8/16
OB|MH546579|mallard|Idaho|H5N2|HA|10/15/16
OB|MK928156|Green-Winged_Teal|Nevada|H5N2|HA|10/11/16
OB|MH546675|mallard|Nevada|H5N2|HA|10/8/16
OB|MH546203|American_green-winged_teal|Nevada|H5N2|HA|10/8/16
OB|MH546571|mallard|California|H5N2|HA|11/19/16
OB|MH546531|lesser_snow_goose|Oregon|H5N3|HA|2/17/16
OB|MH546355|common_murre|Alaska|H5N9|HA|1/5/16
OB|MH546211|American_green-winged_teal|Oregon|H5N2|HA|12/28/16
OB|MH546235|American_wigeon|Oregon|H5N2|HA|12/28/16
OB|MH546723|mallard|Oregon|H5N2|HA|12/28/16
OB|MK995858|Northern_Shoveler|Utah|H5N2|HA|11/14/17
OB|MK995866|Ring-necked_Duck|Arizona|H5N2|HA|11/28/17
OB|MN431106|Mallard|Illinois|H5N2|HA|11/12/17
OB|MH546141|mallard|Alaska|H5N2|HA|9/4/16
OB|MH546971|ring-necked_duck|California|H5N2|HA|1/31/16
OB|CY235315|mallard|Idaho|H5N9|HA|8/18/16
OB|MH546187|American_green-winged_teal|Idaho|H5N9|HA|8/23/16
OB|MH546331|blue-winged_teal|Texas|H5N1|HA|9/17/16
OB|MH546179|American_green-winged_teal|Idaho|H5N9|HA|8/23/16
OB|MH546755|mallard|Utah|H5N9|HA|7/30/16
OB|MH546195|American_green-winged_teal|Idaho|H5N9|HA|8/23/16
OB|MH546707|mallard|Oregon|H5N2|HA|11/8/15
OB|MG280333|gadwall_duck|Tennessee|H5N1|HA|1/14/17
OB|MG279989|black_duck|Tennessee|H5N1|HA|1/13/17
OB|KY284403|gadwall|Arkansas|H5N2|HA|12/18/15
OB|CY213584|sandpiper|Southcentral_Alaska|H5N2|HA|5/9/16
OB|CY213592|sandpiper|Southcentral_Alaska|mixed|HA|5/14/16
OB|CY213518|glaucous-winged_gull|Southcentral_Alaska|H5N2|HA|3/3/16
OB|CY213576|sandpiper|Southcentral_Alaska|H5N2|HA|5/7/16
OB|CY213568|sandpiper|Southcentral_Alaska|H5N2|HA|5/7/16
OB|CY213612|least_sandpiper|Southcentral_Alaska|H5N2|HA|5/11/16
OB|CY213604|least_sandpiper|Southcentral_Alaska|H5N2|HA|5/11/16
OB|MH546243|American_wigeon|Utah|H5N2|HA|11/20/15
OB|MH546219|American_wigeon|California|H5N2|HA|12/2/15
OB|MH546227|American_wigeon|Colorado|H5N2|HA|11/21/15
OB|KY983165|northern_shoveler|California|H5N3|HA|12/5/15
OB|MH546931|northern_shoveler|California|H5N3|HA|12/2/15
OB|MH546587|mallard|Illinois|H5N6|HA|12/1/15
DD|MH546683|mallard|New_Hampshire|H5N3|HA|9/6/16
OB|KY561037|northern_pintail|Ohio|H5N2|HA|11/14/15
OB|MH546467|gadwall|North_Carolina|H5N2|HA|11/14/15
OB|MH546659|mallard|Minnesota|H5N2|HA|9/21/16
OB|MH546651|mallard|Minnesota|H5N2|HA|8/31/16
OB|MH546627|mallard|Minnesota|H5N9|HA|9/18/15
OB|MH546643|mallard|Minnesota|H5N2|HA|9/23/15
OB|MG280562|green-winged_teal|Mississippi|H5N2|HA|12/19/16
OB|MH546699|mallard|North_Dakota|H5N1|HA|10/26/16
OB|MH546667|mallard|Minnesota|H5N5|HA|9/21/16
DD|MH546731|mallard|Pennsylvania|H5N6|HA|9/13/16
OB|MH546523|lesser_snow_goose|Arkansas|H5N2|HA|2/9/16
OB|MH546611|mallard|Michigan|H5N2|HA|10/1/15
OB|MF046544|Canada_goose|Delaware_Bay|H5N1|HA|5/25/16
SB|MH068019|ruddy_turnstone|New_Jersey|H5|HA|5/12/17
OB|MH546595|mallard|Indiana|H5N2|HA|10/22/16
OB|MK237194|mallard|Ohio|H5N2|HA|10/25/17
OB|MG280368|American_wigeon|Ohio|H5N2|HA|10/22/16
DD|MT420977|mallard|Maryland|mixed|HA|11/18/16
OB|KY561242|mallard_duck|Ohio|H5N2|HA|8/28/15
SB|MH068503|ruddy_turnstone|New_Jersey|H5N7|HA|5/22/17
SB|MG891864|ruddy_turnstone|Delaware_Bay|H5N7|HA|5/21/17
SB|MG891857|ruddy_turnstone|Delaware_Bay|mixed.H5|HA|5/20/17
SB|MG930796|ruddy_turnstone|Delaware_Bay|H5N7|HA|5/21/17
SB|MG891882|ruddy_turnstone|Delaware_Bay|H5N7|HA|5/21/17
SB|MH068639|ruddy_turnstone|New_Jersey|H5N7|HA|5/27/17
SB|MH068551|ruddy_turnstone|New_Jersey|H5N7|HA|5/22/17
SB|MH068391|ruddy_turnstone|New_Jersey|H5N2|HA|5/13/17
SB|MH068479|ruddy_turnstone|New_Jersey|H5N2|HA|5/19/17
SB|MH068455|ruddy_turnstone|New_Jersey|H5N2|HA|5/19/17
SB|MH068615|ruddy_turnstone|New_Jersey|H5N2|HA|5/24/17
SB|MH068415|ruddy_turnstone|New_Jersey|H5N2|HA|5/13/17
SB|MH068423|ruddy_turnstone|New_Jersey|H5N2|HA|5/13/17
SB|MH068655|ruddy_turnstone|New_Jersey|H5N7|HA|5/27/17
SB|MH068399|ruddy_turnstone|New_Jersey|H5N2|HA|5/13/17
DD|MH546603|mallard|Maryland|H5N2|HA|12/27/16
PO|MH546403|duck|New_York|H5N2|HA|6/21/16
PO|MH546275|Muscovy_duck|New_Jersey|H5N2|HA|7/1/16
PO|MH546419|duck|New_York|H5N2|HA|6/29/16
PO|MH546283|Muscovy_duck|New_Jersey|H5N2|HA|7/1/16
OB|KU201728|American_green-winged_teal|Idaho|H5N2|HA|1/18/15
OB|MG964760|falcon|Missouri|H5N2|HA|3/1/15
OB|KX838899|mallard|Alaska|H5N2|HA|8/12/16
OB|KU201736|American_green-winged_teal|Oregon|H5N2|HA|1/14/15
OB|KU201943|Canada_goose|Kansas|H5N2|HA|3/13/15
OB|KU201656|wood_duck|Oregon|H5N2|HA|1/3/15
OB|MN037415|coopers_hawk|Washington|H5N2|HA|1/9/15
OB|KU201832|mallard|Washington|H5N2|HA|1/1/15
OB|KU201648|wood_duck|Oregon|H5N2|HA|1/3/15
OB|KU201848|mallard|Washington|H5N2|HA|1/10/15
OB|KU201696|mallard|Oregon|H5N2|HA|1/7/15
OB|KU201712|mallard|Idaho|H5N2|HA|1/17/15
OB|KU201720|mallard|Idaho|H5N2|HA|1/17/15
OB|MG964784|great-horned_owl|Idaho|H5N2|HA|1/21/15
OB|MG964800|peregrine_falcon|Idaho|H5N2|HA|1/9/15
OB|MG964776|great-horned_owl|Idaho|H5N2|HA|1/14/15
OB|KU201624|mallard|Oregon|H5N2|HA|1/5/15
OB|MG964816|ring-necked_duck|Kentucky|H5N2|HA|3/10/15
OB|MG964824|snow_goose|Kentucky|H5N2|HA|3/10/15
OB|MG964360|Canada_goose|Wyoming|H5N2|HA|3/16/15
OB|MG964344|Canada_goose|Michigan|H5N2|HA|6/4/15
OB|KU201936|snowy_owl|Wisconsin|H5N2|HA|4/13/15
OB|KU201928|Coopers_hawk|Minnesota|H5N2|HA|4/14/15
OB|KU201680|Northern_shoveler|Oregon|H5N2|HA|1/3/15
OB|KU201664|Northern_shoveler|Oregon|H5N2|HA|1/3/15
OB|KU201672|Northern_shoveler|Oregon|H5N2|HA|1/3/15
OB|MG964808|red-tailed_hawk|Washington|H5N2|HA|1/9/15
OB|KU201704|Northern_pintail|Oregon|H5N2|HA|1/12/15
OB|KY828643|American_wigeon|California|H5N8|HA|1/17/15
OB|KU201752|American_wigeon|Oregon|H5N8|HA|1/18/15
OB|KU201744|Canada_goose|Oregon|H5N8|HA|1/18/15
OB|KU201856|American_wigeon|Washington|H5N1|HA|1/2/15
OB|KU201864|American_wigeon|Washington|H5N1|HA|1/2/15
OB|KU201616|American_wigeon|Utah|H5N8|HA|1/2/15
OB|KU201760|mallard|Nevada|H5N8|HA|1/23/15
1995
2000
2005
2010
2015
2020

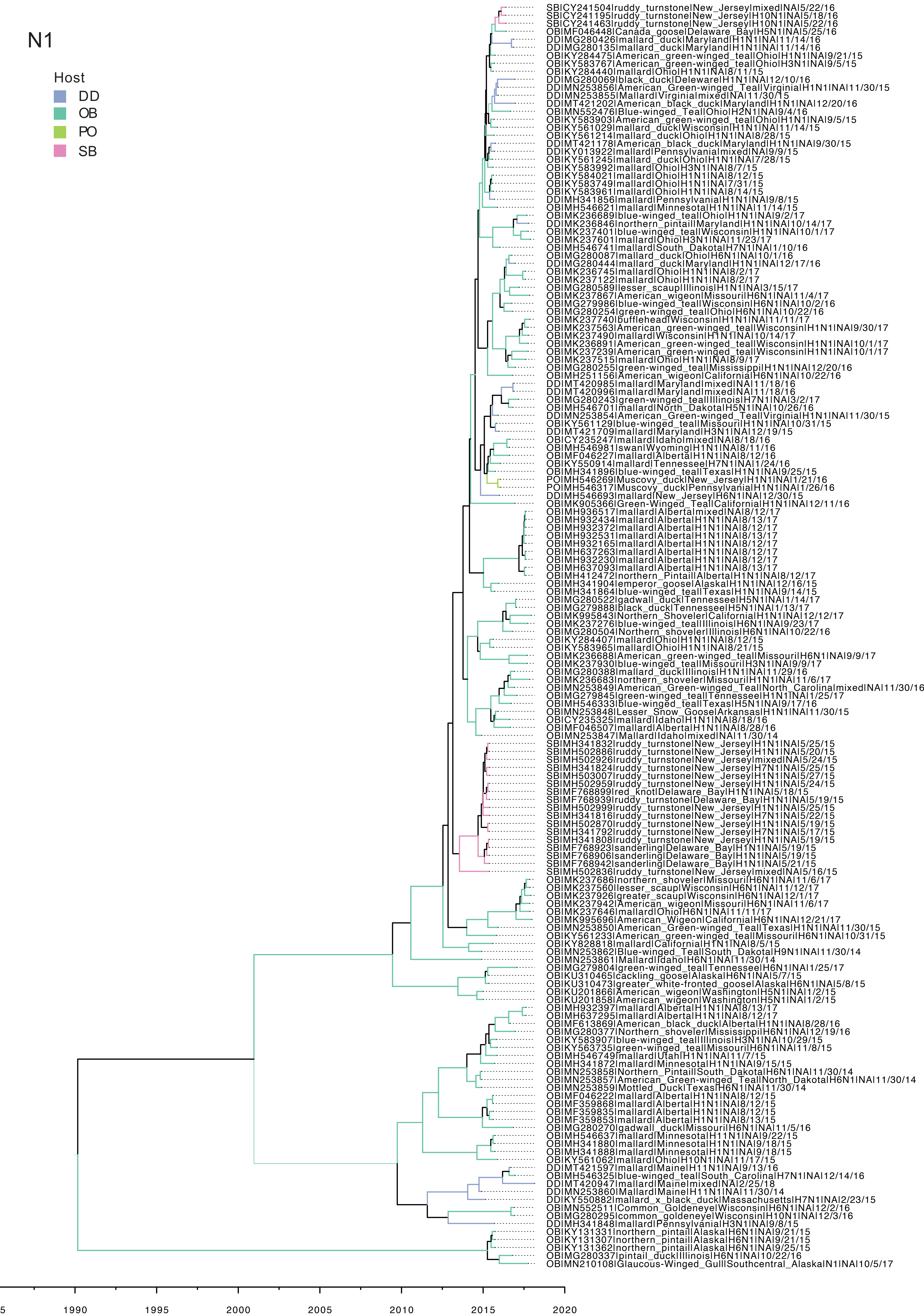

N1
Host
DD
OB
PO
SB
SB|CY241504|ruddy_turnstone|New_Jersey|mixed|NA|5/22/16
SB|CY241195|ruddy_turnstone|New_Jersey|H10N1|NA|5/18/16
SB|CY241463|ruddy_turnstone|New_Jersey|H10N1|NA|5/22/16
OB|MF046448|Canada_goose|Delaware_Bay|H5N1|NA|5/25/16
DD|MG280426|mallard_duck|Maryland|H1N1|NA|11/14/16
DD|MG280135|mallard_duck|Maryland|H1N1|NA|11/14/16
OB|KY284475|American_green-winged_teal|Ohio|H1N1|NA|9/21/15
OB|KY583767|American_green-winged_teal|Ohio|H3N1|NA|9/5/15
OB|KY284440|mallard|Ohio|H1N1|NA|8/11/15
DD|MG280069|black_duck|Delaware|H1N1|NA|12/10/16
DD|MN253856|American_Green-winged_Teal|Virginia|H1N1|NA|11/30/15
DD|MN253855|Mallard|Virginia|mixed|NA|11/30/15
DD|MT421202|American_black_duck|Maryland|H1N1|NA|12/20/16
OB|MN552476|Blue-winged_Teal|Ohio|H2N1|NA|9/4/16
OB|KY583903|American_green-winged_teal|Ohio|H1N1|NA|9/5/15
OB|KY561029|mallard_duck|Wisconsin|H1N1|NA|11/14/15
OB|KY561214|mallard_duck|Ohio|H1N1|NA|8/28/15
DD|MT421178|American_black_duck|Maryland|H1N1|NA|9/30/15
DD|KY013922|mallard|Pennsylvania|mixed|NA|9/9/15
OB|KY561245|mallard_duck|Ohio|H1N1|NA|7/28/15
OB|KY583992|mallard|Ohio|H3N1|NA|8/7/15
OB|KY584021|mallard|Ohio|H1N1|NA|8/12/15
OB|KY583749|mallard|Ohio|H1N1|NA|7/31/15
OB|KY583961|mallard|Ohio|H1N1|NA|8/14/15
DD|MH341856|mallard|Pennsylvania|H1N1|NA|9/8/15
OB|MH546621|mallard|Minnesota|H1N1|NA|11/14/15
OB|MK236689|blue-winged_teal|Ohio|H1N1|NA|9/2/17
DD|MK236846|northern_pintail|Maryland|H1N1|NA|10/14/17
OB|MK237401|blue-winged_teal|Wisconsin|H1N1|NA|10/1/17
OB|MK237601|mallard|Ohio|H3N1|NA|11/23/17
OB|MH546741|mallard|South_Dakota|H7N1|NA|1/10/16
OB|MG280087|mallard_duck|Ohio|H6N1|NA|10/1/16
DD|MG280444|mallard_duck|Maryland|H1N1|NA|12/17/16
OB|MK236745|mallard|Ohio|H1N1|NA|8/2/17
OB|MK237122|mallard|Ohio|H1N1|NA|8/2/17
OB|MG280589|lesser_scaup|Illinois|H1N1|NA|3/15/17
OB|MK237867|American_wigeon|Missouri|H6N1|NA|11/4/17
OB|MG279986|blue-winged_teal|Wisconsin|H6N1|NA|10/2/16
OB|MG280254|green-winged_teal|Ohio|H6N1|NA|10/22/16
OB|MK237740|bufflehead|Wisconsin|H1N1|NA|11/11/17
OB|MK237563|American_green-winged_teal|Wisconsin|H1N1|NA|9/30/17
OB|MK237490|mallard|Wisconsin|H1N1|NA|10/14/17
OB|MK236891|American_green-winged_teal|Wisconsin|H1N1|NA|10/1/17
OB|MK237239|American_green-winged_teal|Wisconsin|H1N1|NA|10/1/17
OB|MK237515|mallard|Ohio|H1N1|NA|8/9/17
OB|MG280255|green-winged_teal|Mississippi|H1N1|NA|12/20/16
OB|MH251156|American_wigeon|California|H6N1|NA|10/22/16
DD|MT420985|mallard|Maryland|mixed|NA|11/18/16
DD|MT420996|mallard|Maryland|mixed|NA|11/18/16
OB|MG280243|green-winged_teal|Illinois|H7N1|NA|3/2/17
OB|MH546701|mallard|North_Dakota|H5N1|NA|10/26/16
DD|MN253854|American_Green-winged_Teal|Virginia|H1N1|NA|11/30/15
OB|KY561129|blue-winged_teal|Missouri|H1N1|NA|10/31/15
DD|MT421709|mallard|Maryland|H3N1|NA|12/19/15
OB|CY235247|mallard|Idaho|mixed|NA|8/18/16
OB|MH546981|swan|Wyoming|H1N1|NA|8/11/16
OB|MF046227|mallard|Alberta|H1N1|NA|8/12/16
OB|KY550914|mallard|Tennessee|H7N1|NA|1/24/16
OB|MH341896|blue-winged_teal|Texas|H1N1|NA|9/25/15
PO|MH546269|Muscovy_duck|New_Jersey|H1N1|NA|1/21/16
PO|MH546317|Muscovy_duck|Pennsylvania|H1N1|NA|1/26/16
DD|MH546693|mallard|New_Jersey|H6N1|NA|12/30/15
OB|MK905366|Green-Winged_Teal|California|H1N1|NA|12/11/16
OB|MH936517|mallard|Alberta|mixed|NA|8/12/17
OB|MH932434|mallard|Alberta|H1N1|NA|8/13/17
OB|MH932372|mallard|Alberta|H1N1|NA|8/12/17
OB|MH932531|mallard|Alberta|H1N1|NA|8/13/17
OB|MH932165|mallard|Alberta|H1N1|NA|8/12/17
OB|MH637263|mallard|Alberta|H1N1|NA|8/12/17
OB|MH932230|mallard|Alberta|H1N1|NA|8/12/17
OB|MH637093|mallard|Alberta|H1N1|NA|8/13/17
OB|MH412472|northern_Pintail|Alberta|H1N1|NA|8/12/17
OB|MH341904|emperor_goose|Alaska|H1N1|NA|12/16/15
OB|MH341864|blue-winged_teal|Texas|H1N1|NA|9/14/15
OB|MG280522|gadwall_duck|Tennessee|H5N1|NA|1/14/17
OB|MG279888|black_duck|Tennessee|H5N1|NA|1/13/17
OB|MK995843|Northern_Shoveler|California|H1N1|NA|12/12/17
OB|MK237276|blue-winged_teal|Illinois|H6N1|NA|9/23/17
OB|MG280504|Northern_shoveler|Illinois|H6N1|NA|10/22/16
OB|KY284407|mallard|Ohio|H1N1|NA|8/12/15
OB|KY583965|mallard|Ohio|H1N1|NA|8/21/15
OB|MK236688|American_green-winged_teal|Missouri|H6N1|NA|9/9/17
OB|MK237930|blue-winged_teal|Missouri|H3N1|NA|9/9/17
OB|MG280388|mallard_duck|Illinois|H1N1|NA|11/29/16
OB|MK236683|northern_shoveler|Missouri|H1N1|NA|11/6/17
OB|MN253849|American_Green-winged_Teal|North_Carolina|mixed|NA|11/30/16
OB|MG279845|green-winged_teal|Tennessee|H1N1|NA|1/25/17
OB|MH546333|blue-winged_teal|Texas|H5N1|NA|9/17/16
OB|MN253848|Lesser_Snow_Goose|Arkansas|H1N1|NA|11/30/15
OB|CY235325|mallard|Idaho|H1N1|NA|8/18/16
OB|MF046507|mallard|Alberta|H1N1|NA|8/28/16
OB|MN253847|Mallard|Idaho|mixed|NA|11/30/14
SB|MH341832|ruddy_turnstone|New_Jersey|H1N1|NA|5/25/15
SB|MH502886|ruddy_turnstone|New_Jersey|H1N1|NA|5/20/15
SB|MH502926|ruddy_turnstone|New_Jersey|mixed|NA|5/24/15
SB|MH341824|ruddy_turnstone|New_Jersey|H7N1|NA|5/25/15
SB|MH503007|ruddy_turnstone|New_Jersey|H1N1|NA|5/27/15
SB|MH502959|ruddy_turnstone|New_Jersey|H1N1|NA|5/24/15
SB|MF768899|red_knot|Delaware_Bay|H1N1|NA|5/18/15
SB|MF768939|ruddy_turnstone|Delaware_Bay|H1N1|NA|5/19/15
SB|MH502999|ruddy_turnstone|New_Jersey|H1N1|NA|5/25/15
SB|MH341816|ruddy_turnstone|New_Jersey|H7N1|NA|5/22/15
SB|MH502870|ruddy_turnstone|New_Jersey|H1N1|NA|5/19/15
SB|MH341792|ruddy_turnstone|New_Jersey|H7N1|NA|5/17/15
SB|MH341808|ruddy_turnstone|New_Jersey|H1N1|NA|5/19/15
SB|MF768923|sanderling|Delaware_Bay|H1N1|NA|5/19/15
SB|MF768906|sanderling|Delaware_Bay|H1N1|NA|5/19/15
SB|MF768942|sanderling|Delaware_Bay|H1N1|NA|5/21/15
SB|MH502836|ruddy_turnstone|New_Jersey|mixed|NA|5/16/15
OB|MK237686|northern_shoveler|Missouri|H6N1|NA|11/6/17
OB|MK237560|lesser_scaup|Wisconsin|H6N1|NA|11/12/17
OB|MK237926|greater_scaup|Wisconsin|H6N1|NA|12/1/17
OB|MK237942|American_wigeon|Missouri|H6N1|NA|11/6/17
OB|MK237646|mallard|Ohio|H6N1|NA|11/11/17
OB|MK995696|American_Wigeon|California|H6N1|NA|12/21/17
OB|MN253850|American_Green-winged_Teal|Texas|H1N1|NA|11/30/15
OB|KY561233|American_green-winged_teal|Missouri|H6N1|NA|10/31/15
OB|KY828818|mallard|California|H1N1|NA|8/5/15
OB|MN253862|Blue-winged_Teal|South_Dakota|H9N1|NA|11/30/14
OB|MN253861|Mallard|Idaho|H6N1|NA|11/30/14
OB|MG279804|green-winged_teal|Tennessee|H6N1|NA|1/25/17
OB|KU310465|cackling_goose|Alaska|H6N1|NA|5/7/15
OB|KU310473|greater_white-fronted_goose|Alaska|H6N1|NA|5/8/15
OB|KU201866|American_wigeon|Washington|H5N1|NA|1/2/15
OB|KU201858|American_wigeon|Washington|H5N1|NA|1/2/15
OB|MH932397|mallard|Alberta|H1N1|NA|8/13/17
OB|MH637295|mallard|Alberta|H1N1|NA|8/12/17
OB|MF613869|American_black_duck|Alberta|H1N1|NA|8/28/16
OB|MG280377|Northern_shoveler|Mississippi|H6N1|NA|12/19/16
OB|KY583907|blue-winged_teal|Illinois|H3N1|NA|10/29/15
OB|KY563735|green-winged_teal|Missouri|H6N1|NA|11/8/15
OB|MH546749|mallard|Utah|H1N1|NA|11/7/15
OB|MH341872|mallard|Minnesota|H1N1|NA|9/15/15
OB|MN253858|Northern_Pintail|South_Dakota|H6N1|NA|11/30/14
OB|MN253857|American_Green-winged_Teal|North_Dakota|H6N1|NA|11/30/14
OB|MN253859|Mottled_Duck|Texas|H6N1|NA|11/30/14
OB|MF046222|mallard|Alberta|H1N1|NA|8/12/15
OB|MF359868|mallard|Alberta|H1N1|NA|8/12/15
OB|MF359835|mallard|Alberta|H1N1|NA|8/12/15
OB|MF359853|mallard|Alberta|H1N1|NA|8/13/15
OB|MG280270|gadwall_duck|Missouri|H6N1|NA|11/5/16
OB|MH546637|mallard|Minnesota|H11N1|NA|9/22/15
OB|MH341880|mallard|Minnesota|H1N1|NA|9/18/15
OB|MH341888|mallard|Minnesota|H1N1|NA|9/18/15
OB|KY561062|mallard|Ohio|H10N1|NA|11/17/15
DD|MT421597|mallard|Maine|H11N1|NA|9/13/16
OB|MH546325|blue-winged_teal|South_Carolina|H7N1|NA|12/14/16
DD|MT420947|mallard|Maine|mixed|NA|2/25/18
DD|MN253860|Mallard|Maine|H11N1|NA|11/30/14
DD|KY550882|mallard_x_black_duck|Massachusetts|H7N1|NA|2/23/15
OB|MN552511|Common_Goldeneye|Wisconsin|H6N1|NA|12/2/16
OB|MG280295|common_goldeneye|Wisconsin|H10N1|NA|12/3/16
DD|MH341848|mallard|Pennsylvania|H3N1|NA|9/8/15
OB|KY131331|northern_pintail|Alaska|H6N1|NA|9/21/15
OB|KY131307|northern_pintail|Alaska|H6N1|NA|9/21/15
OB|KY131362|northern_pintail|Alaska|H6N1|NA|9/25/15
OB|MG280337|pintail_duck|Illinois|H6N1|NA|10/22/16
OB|MN210108|Glaucous-Winged_Gull|Southcentral_Alaska|N1|NA|10/5/17
5
1990
1995
2000
2005
2010
2015
2020

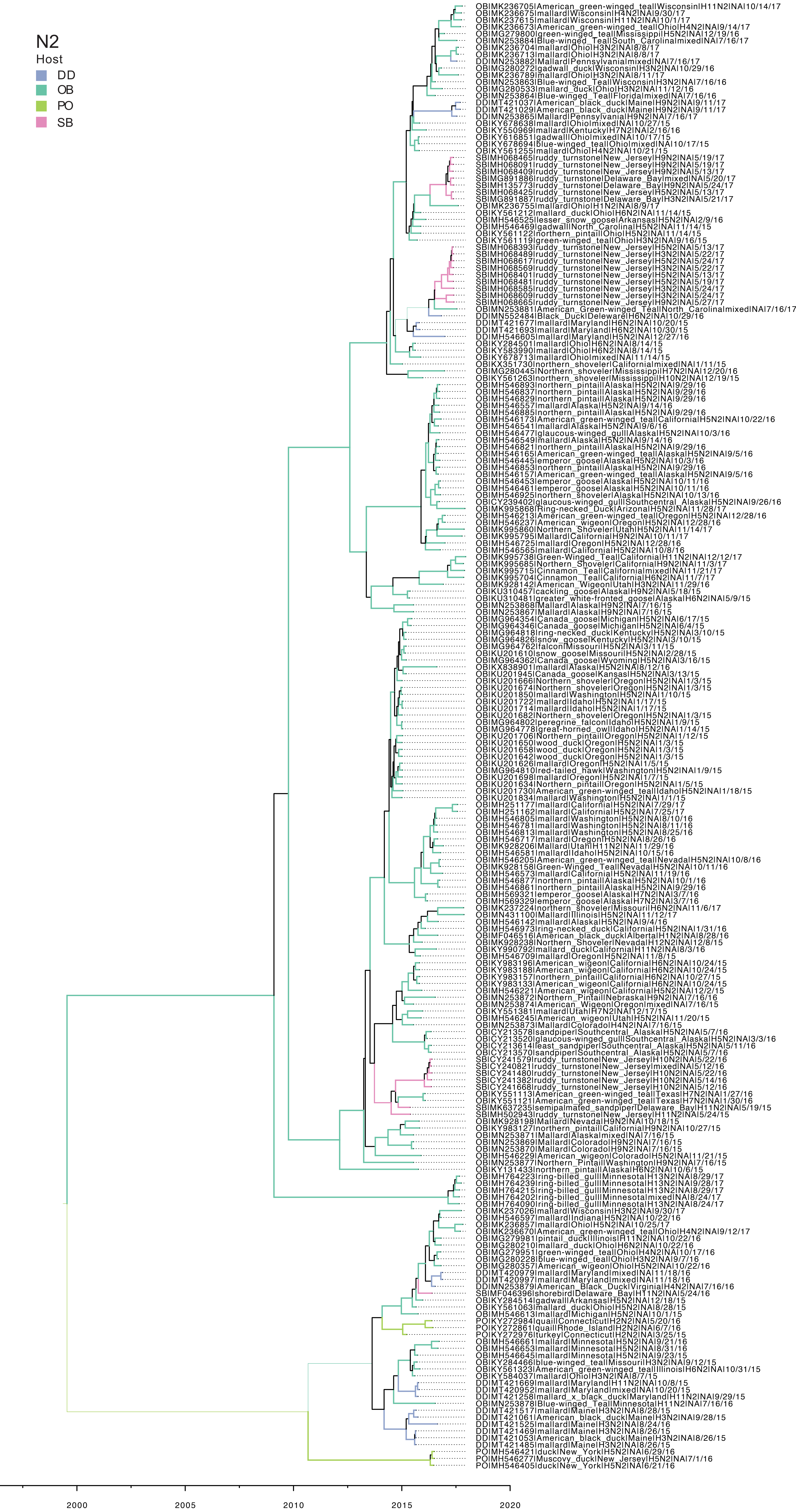
N2
Host
DD
OB
PO
SB
2000
2005
2010
2015
2020
